# Antibody–drug conjugates in advanced small-cell lung cancer: a systematic review and single-arm meta-analysis of later-line monotherapy

**DOI:** 10.3389/fimmu.2026.1929911

**Published:** 2026-10-07

**Authors:** Xinmiao Chen, Shaoxun Li, Huiying Li

**Affiliations:** 1 Department of Respiratory and Critical Care Medicine, The Second Affiliated Hospital and Yuying Children’s Hospital of Wenzhou Medical University, Wenzhou, Zhejiang, China; 2 Department of Infectious Disease, The Second Affiliated Hospital and Yuying Children’s Hospital of Wenzhou Medical University, Wenzhou, Zhejiang, China

**Keywords:** antibody–drug conjugate, generalized linear mixed model, single-arm meta-analysis, small-cell lung cancer, systematic review

## Abstract

**Objective:**

Antibody–drug conjugates (ADCs) are being investigated in advanced small-cell lung cancer (SCLC), but the evidence is dominated by early-phase studies conducted in non-exchangeable treatment settings. We evaluated the activity and toxicity of ADCs while separating later-line monotherapy from frontline combination therapy, later-line combination therapy, and post-induction maintenance.

**Methods:**

PubMed, Embase, the Cochrane Library, and Web of Science Core Collection were searched from inception and in 28 August 2026. ClinicalTrials.gov and the WHO International Clinical Trials Registry Platform (ICTRP) were searched on 28 August 2026. We constructed an arm-level dataset that recorded regimen, dose, clinical context, target, outcome definition, numerator, denominator, analysis set, and source. The primary quantitative synthesis was restricted to second-line or later ADC monotherapy; other clinical contexts were summarized descriptively. Single-arm proportions were synthesized using a binomial–normal random-intercept generalized linear mixed model on the logit scale. We reported 95% confidence intervals (CIs), between-study variance, heterogeneity, and 95% prediction intervals. An inverse-variance logit random-effects model with restricted maximum likelihood estimation and Hartung–Knapp inference was used as a sensitivity analysis. Fixed-time Kaplan–Meier survival estimates and inconsistently defined common adverse events were not pooled.

**Results:**

16 trial families evaluating eight ADCs across five target classes met the eligibility criteria. In the primary later-line monotherapy analysis, the pooled ORR was 25.7% (95% CI, 16.4%–37.9%), and the pooled DCR was 72.7% (95% CI, 60.7%–82.2%). The pooled incidences of grade ≥3 TEAEs and grade ≥3 TRAEs were 62.8% (95% CI, 59.6%–65.9%) and 45.5% (95% CI, 35.5%–56.0%), respectively. The prediction intervals for ORR, DCR, and grade ≥3 TRAEs were wide. Proportions observed in a new study may therefore differ substantially from the pooled estimates. Frontline combinations, later-line combinations, and maintenance therapy yielded context-specific signals but were not compared with later-line monotherapy. Target-stratified analyses were exploratory; B7-H3 and DLL3 estimates were based on small, non-comparative groups, whereas Trop-2 and SEZ6 were not pooled because fewer than three studies were available.

**Conclusions:**

Later-line ADC monotherapy showed antitumor activity but frequent clinically important toxicity, with substantial between-study variation. The evidence does not establish superiority by treatment line, regimen, agent, or molecular target; single-arm response estimates also do not demonstrate a survival benefit. Randomized, context-specific trials using harmonized efficacy and safety definitions are needed.

## 1 Introduction

Small-cell lung cancer (SCLC) is an aggressive neuroendocrine malignancy characterized by rapid growth, early dissemination, and frequent relapse. Although the addition of a programmed death-ligand 1 inhibitor to platinum–etoposide has changed first-line treatment for extensive-stage disease, long-term survival remains limited, and therapeutic options after progression are inadequate (1–5).

Antibody–drug conjugates (ADCs) combine a target-specific monoclonal antibody with a cytotoxic payload through a linker, enabling selective payload delivery and, for some constructs, a bystander effect (6–11). Multiple ADC programs have entered clinical testing in SCLC, including agents directed against delta-like ligand 3 (DLL3), B7-H3, trophoblast cell-surface antigen 2 (Trop-2), seizure-related homolog 6 (SEZ6), and CD56 (12–27).

The evidence is difficult to interpret across studies. Most reports are early-phase and single-arm, and they differ in treatment line, monotherapy versus combination use, maintenance timing, ADC construct, dose, eligibility criteria, outcome definitions, and analysis-set denominators. Pooling these non-exchangeable contexts or treating target groups as comparative categories can produce clinically misleading summaries.

We therefore performed a systematic review and context-specific single-arm meta-analysis. The primary quantitative estimand was the activity and toxicity observed with second-line or later ADC monotherapy. Frontline combinations, later-line combinations, and post-induction maintenance were summarized separately. Exploratory target-stratified analyses were conducted without between-target testing or ranking. Outcome-specific numerators and denominators were verified, and overlapping reports and dependent cohorts were resolved before synthesis.

## 2 Methods

### 2.1 Data sources and search strategy

The review was prospectively registered in PROSPERO on 30 March 2026 (CRD420261354772). We searched PubMed, Embase, the Cochrane Library, and Web of Science Core Collection from inception, with the searches updated in 28 August 2026. The strategies combined controlled vocabulary and free-text terms for small-cell lung cancer with generic antibody–drug conjugate and immunoconjugate terminology and agent-specific names. No study-design filter was applied because eligible evidence included early-phase and single-arm clinical studies. A target-enriched sensitivity search was also conducted, and no language restrictions were applied. Complete platform-specific strategies, dates, and stepwise retrieval counts are provided in **Supplementary Table S1**.

ClinicalTrials.gov and the WHO ICTRP were additionally searched on 28 August 2026 using terms for small-cell lung cancer and antibody-drug conjugates, including agent-specific terms. Conference websites and proceedings, together with the reference lists of included studies and relevant reviews, were also searched. Search results were deduplicated before title and abstract screening, and potentially eligible reports underwent full-text assessment.

Two investigators independently conducted the literature search, record export, deduplication, and initial screening. Disagreements were resolved by discussion, with adjudication by a third investigator when required.

### 2.2 Eligibility criteria and clinical-context classification

We included clinical trials enrolling adults with histologically or cytologically confirmed advanced SCLC who received an ADC either as monotherapy or as part of an ADC-containing regimen. Eligible studies were required to report at least one prespecified efficacy or safety outcome. Reviews, meta-analyses, case reports, preclinical studies, and reports without an extractable SCLC outcome were excluded. Multiple reports from the same trial family were linked before analysis. When reports overlapped, the most complete peer-reviewed report was used for the relevant outcome, with supplementary or earlier reports used only to clarify data that were not duplicated.

Before quantitative synthesis, each ADC arm or cohort was classified by clinical context as (1) frontline combination therapy, (2) second-line or later monotherapy, (3) second-line or later combination therapy, (4) post-induction maintenance, or (5) mixed or otherwise non-separable context. The primary meta-analytic estimand was the study-level proportion observed with second-line or later ADC monotherapy. Frontline combinations, later-line combinations, and maintenance regimens were not assumed to be exchangeable with later-line monotherapy and were therefore summarized separately. Control arms from randomized trials were excluded from the single-arm proportion meta-analysis; their comparative findings were retained for context and interpreted according to the original randomized comparison.

Studies that combined SCLC with another histological group were eligible for the qualitative review, but they contributed to the SCLC meta-analysis only when SCLC-specific numerators and denominators could be recovered. Accordingly, the SC-002 cohort, which combined SCLC and large-cell neuroendocrine carcinoma without separable outcome data, was not included in the quantitative SCLC synthesis.

### 2.3 Data extraction, analysis sets, and trial-family reconciliation

Two reviewers independently extracted data into a standardized arm-level structure. For each record, we captured the trial identifier, publication, ADC, molecular target, regimen, dose, clinical context, treatment arm or cohort, outcome name and definition, event count, denominator, analysis-set label, source file, source location, and reason for inclusion or exclusion from each synthesis. Disagreements were resolved by discussion, with adjudication by a third reviewer when required.

Numerators and denominators were linked to the outcome-specific analysis set reported by the source. Efficacy and safety denominators were not assumed to be interchangeable. For studies reporting multiple doses or regimens, disjoint cohorts with the same clinical context and compatible outcome definitions were combined at the study level by summing events and denominators before meta-analysis. When a dose-specific cohort was nested within a larger cohort, only the non-duplicative overall cohort was retained for that outcome. Cohorts that differed materially in treatment context or that could not be shown to be independent were not combined. Each trial family contributed only once to a given pooled outcome.

The 2021 peer-reviewed full report by Calvo et al. was used in place of the 2019 conference abstract from the same trial family. The abstract and full report were not counted as separate studies. All reconciled records, including excluded control arms, superseded reports, nested cohorts, and non-separable populations, are documented in the supplementary arm-level dataset with an explicit exclusion reason.

### 2.4 Outcomes

The principal efficacy outcome was ORR, defined as the proportion of patients with a complete or partial response according to the study-reported response criteria. DCR, defined as complete response, partial response, or stable disease, was a secondary efficacy outcome. When a source used a duration-restricted clinical-benefit definition, that value was not treated as DCR unless the components required for the prespecified DCR definition could be reconstructed directly.

The principal safety outcomes were grade ≥3 TEAEs and grade ≥3 TRAEs. Any-grade and serious TEAEs and TRAEs were evaluated as secondary safety outcomes when attribution and grading were reported clearly. TEAEs and TRAEs were retained as distinct estimands because investigator attribution and reporting rules differed across studies.

Landmark PFS and OS values were extracted as reported for descriptive purposes. They were not pooled because the available publications did not provide censoring-aware standard errors or confidence intervals consistently, and Kaplan–Meier probabilities or numbers at risk cannot be analyzed as ordinary binomial event counts. Study-reported common adverse events were also summarized without meta-analysis because attribution rules, reporting thresholds, regimen contexts, and outcome labels were not sufficiently consistent to support a common pooled estimand.

### 2.5 Methodological quality and risk of bias

Non-randomized and single-arm studies were appraised using the Methodological Index for Non-Randomized Studies (MINORS). The item-level scores and prespecified descriptive categories are reported in **Supplementary Table S4**. Randomized trials were assessed with the Cochrane Risk of Bias 2 tool for explicitly defined comparative results: overall survival at the final TAHOE analysis, overall survival in DLL3-high patients at the protocol-defined MERU futility analysis, and progression-free survival at the final IMGN901 phase 2 analysis. The estimand was the effect of assignment to intervention. Judgments and domain rationales were based on the published reports; unavailable protocols or statistical analysis plans were not presumed. These RoB 2 judgments do not constitute an assessment of bias in the single-arm proportions extracted from the ADC groups. Methodological appraisal was kept distinct from certainty of evidence. Because a formal GRADE assessment or an equivalent outcome-level certainty framework was not performed, no certainty rating was assigned to the pooled estimates.

To address potential bias in the absolute ADC-arm proportions directly, we also recorded structured, outcome-level considerations for every cohort contributing to the primary ORR, DCR, or safety synthesis. The domains covered patient selection; the analysis population and missing outcomes; response ascertainment; outcome definition and assessment timing; selective reporting; and dose- or cohort-level representativeness. This descriptive assessment was based on the published reports, was not converted to an overall score, and was kept separate from certainty of evidence (**Supplementary Table S9**).

### 2.6 Statistical analysis

All analyses were conducted in R version 4.4.0 using the meta (version 8.1-0) and metafor (version 4.8-0) packages. The unit of analysis was an independent study-level ADC arm or cohort after resolution of nested and disjoint dose groups. Study-specific proportions were accompanied by exact two-sided 95% Clopper–Pearson CIs.

For outcomes reported by at least three independent later-line monotherapy studies, the primary synthesis used a binomial–normal random-intercept generalized linear mixed model on the logit scale. The model was fitted by maximum likelihood and did not require a continuity correction. Model-based 95% CIs used a classical Wald z approximation on the logit scale and were back-transformed to the proportion scale. We also reported the between-study variance on the logit scale (τ^2^), I^2^, and a 95% prediction interval calculated with the Veroniki method V, using a t distribution with k−1 degrees of freedom, as implemented in the meta package. The prediction interval was used to describe the range of underlying proportions compatible with the model for a future comparable study; it was not interpreted as a treatment-effect interval relative to a control group.

As a model-based sensitivity analysis, we fitted an inverse-variance random-effects model to logit-transformed proportions, estimated τ^2^ by restricted maximum likelihood, and used Hartung–Knapp inference for the pooled CI. A continuity correction of 0.5 was applied only to zero-event or all-event boundary proportions. Prediction intervals in this sensitivity model also used the Veroniki method V. Forest plots display the exact study-level proportions and CIs, ADC arm labels, event counts and denominators, and inverse-variance random-effects weights from the sensitivity model. Square area is proportional to the displayed weight. The displayed weights are from the sensitivity model; conventional study weights are not defined for the primary GLMM.

Leave-one-study-out analyses were performed for outcomes represented by at least four studies. Their purpose was to characterize the influence of individual studies on the summary proportion and heterogeneity, not to establish robustness. Frontline combination, later-line combination, and maintenance data were presented as study-specific exact proportions. Target-stratified analyses were restricted to later-line monotherapy, considered exploratory, and pooled only when at least three independent studies were available. We did not perform between-target tests, meta-regression, or treatment rankings because the small number of studies and confounding by agent, payload, dose, study era, prior therapy, and patient selection made such comparisons unreliable.

Funnel plots and Egger or Begg tests were not performed. Most outcome syntheses contained too few studies for informative asymmetry assessment, and conventional small-study-effect procedures are difficult to interpret for sparse, heterogeneous single-arm proportions. The possibility of selective publication therefore remained unresolved. All 95% intervals were two-sided.

## 3 Results

### 3.1 Study characteristics and clinical contexts

The searches identified 7, 193 records from bibliographic databases and 85 records from trial registers. After removal of 3, 314 duplicate records, 3, 964 records underwent title and abstract screening, of which 3, 813 were excluded. The full texts of 151 reports were sought and successfully retrieved, and all were assessed for eligibility. Of these, 135 reports were excluded because of ineligible populations (n = 53), ineligible interventions (n = 65), or because they were secondary reports (n = 17). Supplementary searches identified an additional 57 records through websites (n = 50) and citation searching (n = 7); no records were identified through organizations or other methods. All 57 reports were retrieved and assessed for eligibility and were subsequently excluded because of ineligible populations (n = 32), ineligible interventions (n = 18), or because they were secondary reports (n = 7). No additional eligible studies were identified through these supplementary searches. Overall, 16 studies, represented by 16 reports, were included in the systematic review (12–27) (**Figure 1**). The included evidence was published between 2017 and 2026 and evaluated eight ADCs directed at five target classes: B7-H3, DLL3, Trop-2, SEZ6, and CD56. Three reports described randomized trials, and the remaining studies were non-randomized or single-arm. The studies differed in developmental phase, ADC structure, payload, dose, biomarker selection, prior treatment exposure, and outcome analysis set.

**Figure 1:**
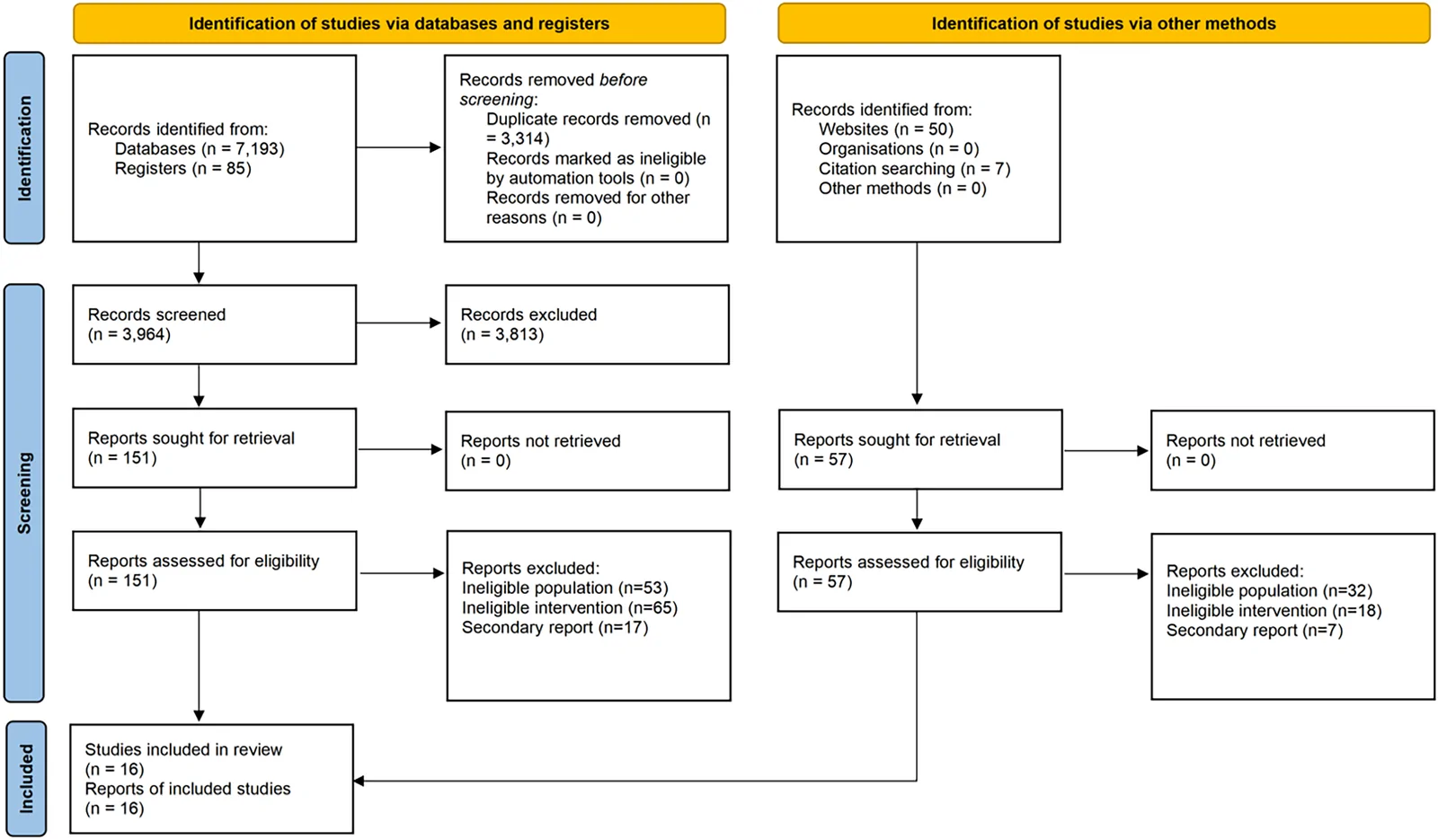
PRISMA 2020 flow diagram of study identification and selection.

The eligible cohorts represented clinically distinct settings. Quantitative synthesis was therefore restricted to second-line or later ADC monotherapy. Frontline ADC–chemotherapy regimens, later-line ADC–immunotherapy combinations, and post-induction maintenance were retained as separate descriptive evidence. **Table 1** presents trial and regimen characteristics by context, and **Supplementary Tables S2A**, **S2B** link every outcome to its numerator, denominator, analysis set, source, and reconciliation details.

**Table 1:** Characteristics of included trial families and ADC cohorts, classified by clinical context.

Study/report	Trial identifier	Design/phase	Clinical context	ADC regimen	Target	Efficacy analysis set	Safety analysis set	Synthesis status
Ma et al., 2025 (12)	NCT05434234; NCT06057922	Phase 1/1b; non-randomized	Later-line monotherapy	YL201, 0.8–3.0 mg/kg Q3W	B7-H3	n=72; recommended-dose n=67	n=79; recommended-dose n=74	Included; outcome-specific sets
Dowlati et al., 2025 (13)	TROPiCS-03; NCT03964727	Phase 2; non-randomized	Second-line monotherapy	Sacituzumab govitecan, 10 mg/kg days 1 and 8 Q21D	Trop-2	n=43	n=43	Included
Rudin et al., 2026 (14)	IDeate-Lung01; NCT05280470	Phase 2; dose optimization/expansion	Later-line monotherapy	Ifinatamab deruxtecan, 8 or 12 mg/kg Q3W	B7-H3	n=183; 12-mg/kg n=137	n=183; 12-mg/kg n=137	Included; disjoint doses combined
Wang et al., 2024 (16)	ARTEMIS-001; NCT05276609	Phase 1a/b; non-randomized	Later-line monotherapy	HS-20093, 8 or 10 mg/kg Q3W	B7-H3	n=52	n=56	Efficacy included; safety counts unavailable
Morgensztern et al., 2024 (15)	NCT03639194	Phase 1; non-randomized	Later-line monotherapy	ABBV-011, 0.3–2.0 mg/kg Q3W	SEZ6	Overall n=98; 1-mg/kg n=40	Overall n=99; 1-mg/kg n=40	Included; nested subsets not added
Blackhall et al., 2021 (17)	TAHOE; NCT03061812	Phase 3; randomized	Second-line monotherapy	Rovalpituzumab tesirine, 0.3 mg/kg Q42D	DLL3	ADC arm n=287	ADC arm n=287	ADC arm included; control excluded
Morgensztern et al., 2020 (18)	NCT02500914	Phase 1; non-randomized	Later-line mixed histology	SC-002, 0.025–0.4 mg/kg Q3W	DLL3	SCLC/LCNEC combined n=35	SCLC/LCNEC combined n=35	Qualitative only; histology inseparable
Morgensztern et al., 2019 (19)	TRINITY; NCT02674568	Phase 2; non-randomized	Third-line or later monotherapy	Rovalpituzumab tesirine, 0.3 mg/kg Q6W	DLL3	n=339	n=339	Included; non-harmonizable grade ≥3 TRAE excluded
Calvo et al., 2021 (20)	NCT03000257	Phase 1; non-randomized	Later-line combination	Rovalpituzumab tesirine + budigalimab	DLL3	n=29	n=31	Descriptive; 2019 abstract superseded
Rudin et al., 2017 (21)	NCT01901653	Phase 1; non-randomized	Later-line monotherapy	Rovalpituzumab tesirine, active-dose schedules	DLL3	n=60	n=74	Included; endpoint-specific sets
Gray et al., 2017 (22)	NCT01631552	Phase 1/2; non-randomized	Later-line monotherapy	Sacituzumab govitecan, 8 or 10 mg/kg	Trop-2	n=50	n=53	Included; DCR reconstructed from best response
Johnson et al., 2021 (23)	MERU; NCT03033511	Phase 3; randomized	Post-induction maintenance	Rovalpituzumab tesirine, 0.3 mg/kg Q6W	DLL3	ADC arm n=318	ADC arm n=368	Descriptive; control excluded
Socinski et al., 2017 (24)	NCT01237678	Phase 1/2; randomized	Frontline combination	Lorvotuzumab mertansine + carboplatin/etoposide	CD56	ADC-combination arm n=82	ADC-combination arm n=94	Descriptive; control excluded
Hann et al., 2021 (25)	NCT02819999	Phase 1; non-randomized	Frontline combination	Rovalpituzumab tesirine + concurrent platinum/etoposide	DLL3	Cohort 3 n=14	Cohort 3 n=14	Descriptive; mixed cohorts separated
Malhotra et al., 2021 (26)	NCT03026166	Phase 1/2; non-randomized	Later-line combination	Rovalpituzumab tesirine + nivolumab ± ipilimumab	DLL3	n=40	n=42	Descriptive
Udagawa et al., 2019 (27)	NCT03086239	Phase 1; non-randomized	Later-line monotherapy	Rovalpituzumab tesirine, 0.2 or 0.3 mg/kg Q6W	DLL3	n=29	n=29	Included

ADC, antibody–drug conjugate; DCR, disease control rate; LCNEC, large-cell neuroendocrine carcinoma; ORR, objective response rate; Q3W, every 3 weeks; Q6W, every 6 weeks; Q21D, every 21 days; Q42D, every 42 days. Analysis sets are outcome specific. Control arms are excluded from the single-arm ADC proportion synthesis.

### 3.2 Data reconciliation and analytical cohorts

Outcome-specific denominators frequently differed within the same study because response and safety populations were defined separately. The reanalysis used the denominator stated for each outcome rather than a single nominal trial sample size. Disjoint dose cohorts with compatible definitions were combined once at the study level, whereas nested cohorts were not counted twice. The Calvo et al., 2021 full report replaced the earlier conference abstract from the same trial family. The SC-002 study was retained in the qualitative evidence map but excluded from the quantitative SCLC analysis because its SCLC and large-cell neuroendocrine carcinoma outcomes were inseparable. Details of these decisions are provided in **Supplementary Table S3**.

### 3.3 Methodological quality and risk of bias

Among the 13 non-randomized or single-arm studies assessed with MINORS, seven met the prespecified high-category threshold and six were in the intermediate category; none was in the low category. These scores describe the reporting and methodological features captured by MINORS and should not be interpreted as certainty of evidence. In the report-based, result-specific RoB 2 assessment, the TAHOE overall-survival result and the MERU DLL3-high overall-survival result raised some concerns, while the IMGN901 progression-free-survival result was judged at high risk because multiple domains raised concerns, including the postbaseline efficacy-set restriction, open-label outcome assessment, protocol amendments, and incomplete reporting of the comparative analysis plan (**Supplementary Table S5**; **Supplementary Figure S1**). These judgments apply to the stated randomized comparisons, not to the single-arm proportions in the primary meta-analysis.

The separate assessment of cohorts contributing absolute ADC-arm proportions identified recurring concerns about restrictive early-phase eligibility, response-evaluable rather than all-treated denominators in some studies, investigator-based response assessment, incomplete verification of prespecified analyses, and the combination of dose-escalation and expansion cohorts. These considerations are reported by domain without an overall rating in **Supplementary Table S9**.

### 3.4 Efficacy of later-line ADC monotherapy

#### 3.4.1 Objective response rate

Ten independent later-line monotherapy cohorts contributed ORR data. Across these cohorts, 291 of 1213 response-evaluable patients had an objective response. The GLMM summary proportion was 25.7% (95% CI, 16.4%–37.9%), with τ^2^ = 0.746, I^2^ = 93.8%, and a 95% prediction interval of 4.2%–73.1% (**Figure 2**). The wide prediction interval indicates that the response proportion in a new, otherwise comparable study could differ markedly from the average estimate. Accordingly, the pooled value is best interpreted as a descriptive mean across heterogeneous ADC programs rather than an expected response rate for any specific drug.

**Figure 2:**
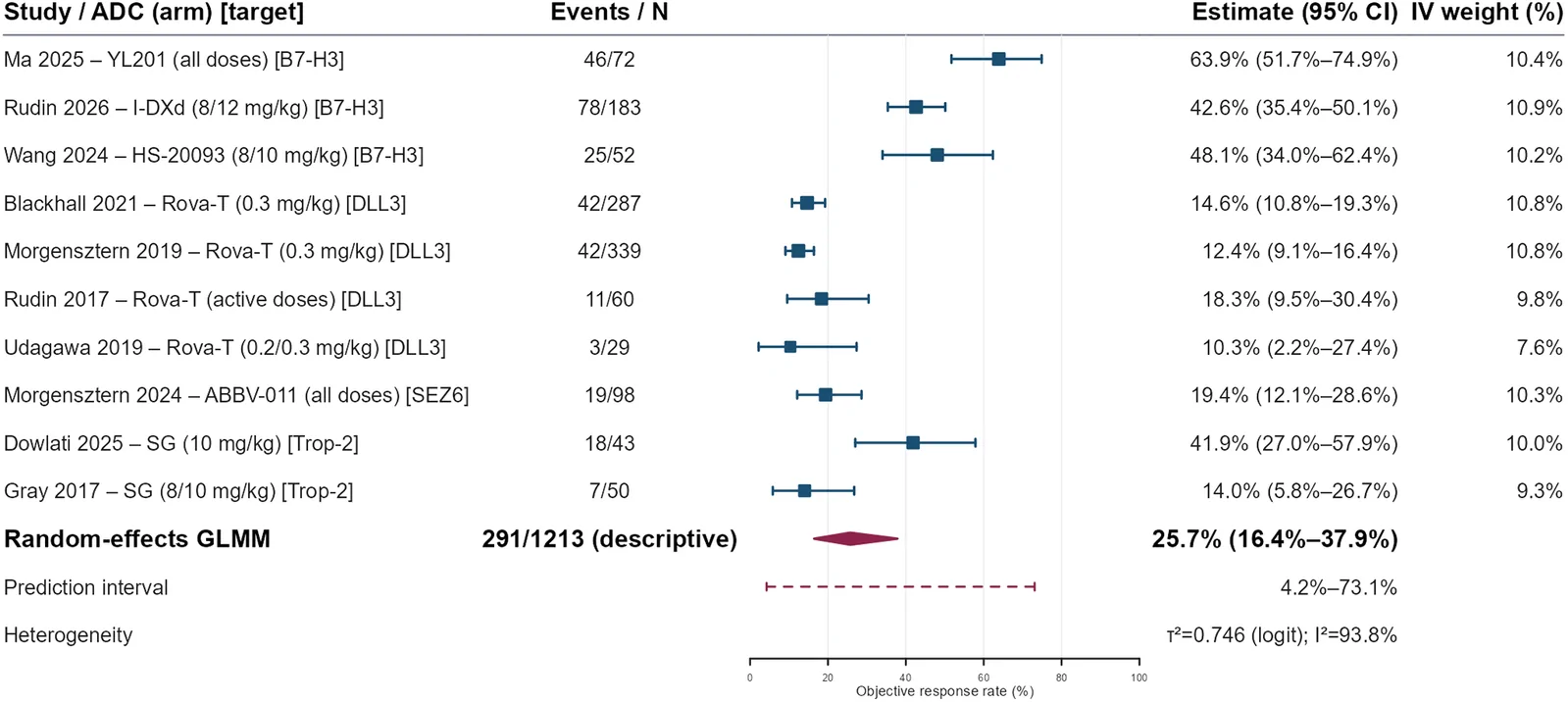
Study-level objective response rates for later-line ADC monotherapy. Squares show study proportions, with area proportional to the inverse-variance logit random-effects weight from the REML sensitivity model; the displayed IV sensitivity weights are not weights from the primary GLMM. Navy horizontal lines are exact 95% Clopper–Pearson confidence intervals. The burgundy diamond shows the primary binomial–normal GLMM summary and its classical Wald z 95% confidence interval; the thin dashed burgundy line below it shows the 95% prediction interval calculated using the Veroniki method V with a t distribution and k−1 degrees of freedom. The sum of events and denominators is descriptive and is not the pooled estimate. τ^2^ is reported on the logit scale.

#### 3.4.2 Disease control rate

Ten independent later-line monotherapy cohorts reported DCR. Together, they included 796 events among 1213 response-evaluable patients. The pooled DCR was 72.7% (95% CI, 60.7%–82.2%), with τ^2^ = 0.691, I^2^ = 94.5%, and a 95% prediction interval of 26.8%–95.1% (**Figure 3**). Between-study dispersion remained substantial. DCR also depended on each trial’s assessment schedule and definition of stable disease; it was therefore not treated as a direct surrogate for durable clinical benefit.

**Figure 3:**
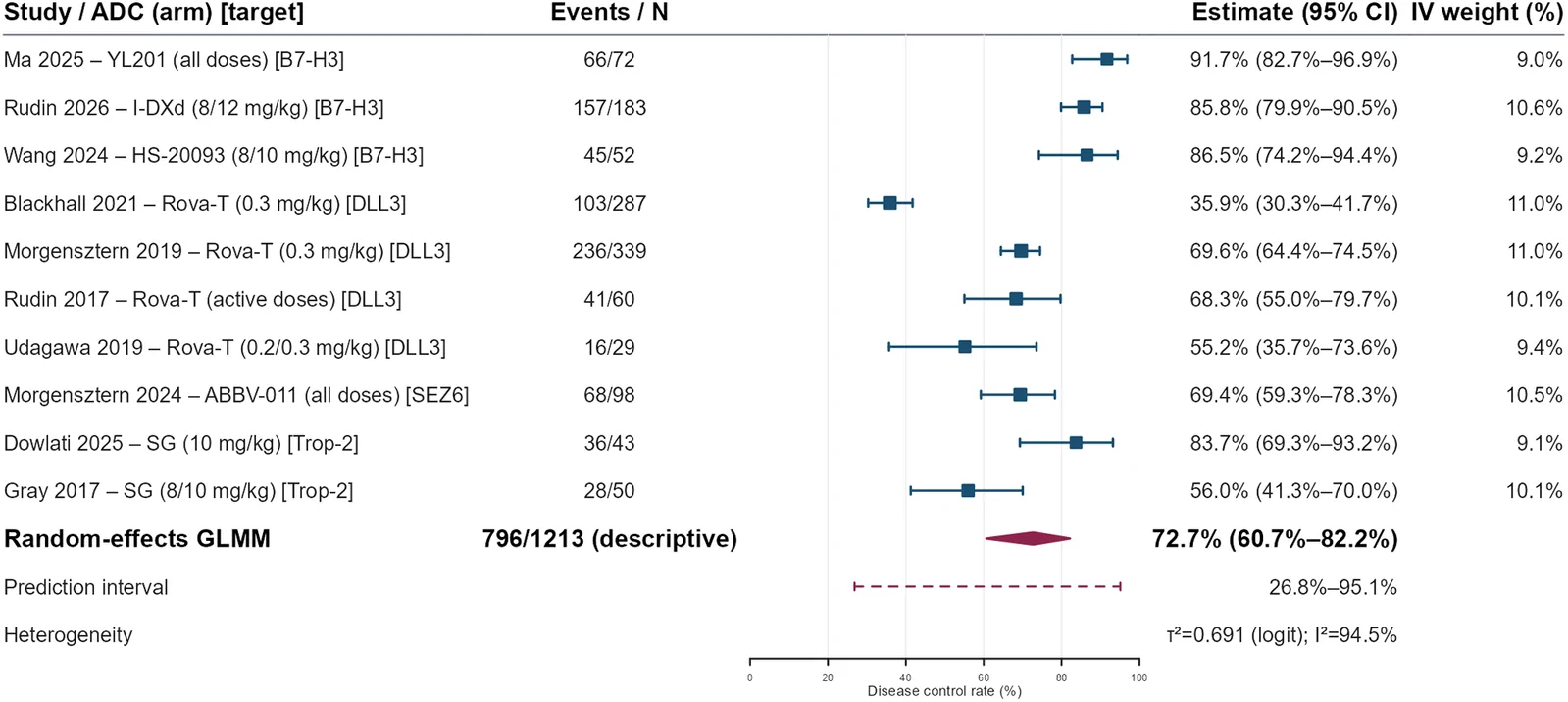
Study-level disease control rates for later-line ADC monotherapy. Squares show study proportions, with area proportional to the inverse-variance logit random-effects weight from the REML sensitivity model; the displayed IV sensitivity weights are not weights from the primary GLMM. Navy horizontal lines are exact 95% Clopper–Pearson confidence intervals. The burgundy diamond shows the primary binomial–normal GLMM summary and its classical Wald z 95% confidence interval; the thin dashed burgundy line below it shows the 95% prediction interval. τ^2^ is reported on the logit scale.

### 3.5 Safety of later-line ADC monotherapy

Five later-line monotherapy cohorts contributed grade ≥3 TEAE data. The pooled incidence was 62.8% (95% CI, 59.6%–65.9%; τ^2^ = 0.000; I^2^ = 10.6%; 95% prediction interval, 58.2%–67.2%) (**Figure 4**). Six cohorts contributed grade ≥3 TRAE data, with a pooled incidence of 45.5% (95% CI, 35.5%–56.0%; τ^2^ = 0.198; I^2^ = 81.8%; 95% prediction interval, 19.0%–74.8%) (**Figure 5**). The distinction between TEAEs and investigator-attributed TRAEs was retained throughout the analysis.

**Figure 4:**
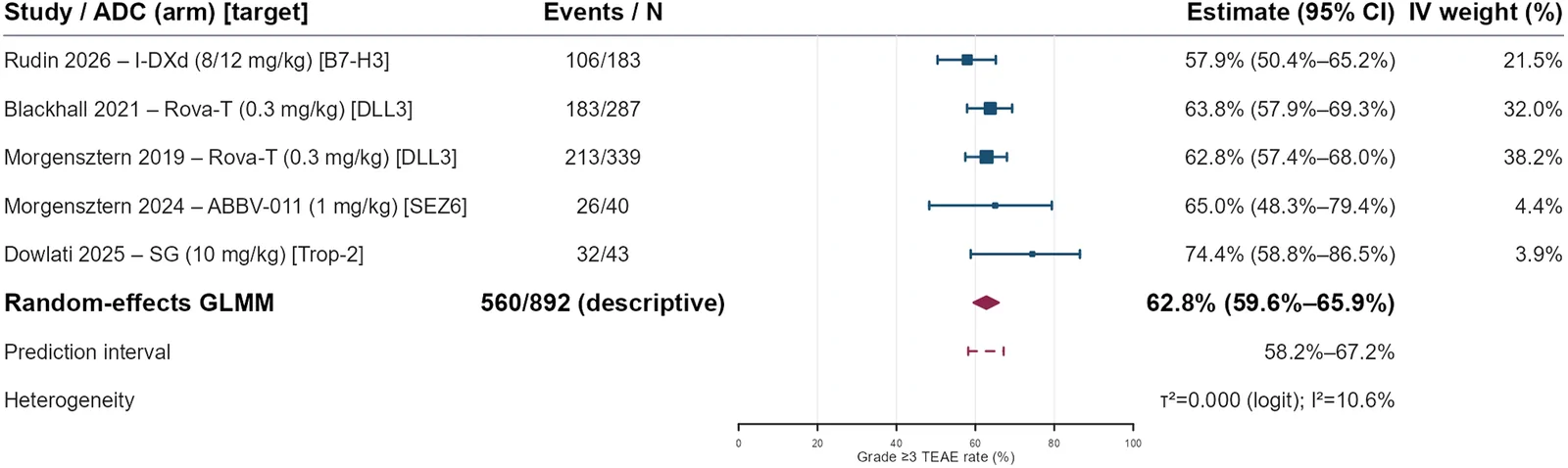
Study-level incidence of grade ≥3 treatment-emergent adverse events for later-line ADC monotherapy. Squares show study proportions, with area proportional to the inverse-variance logit random-effects weight from the REML sensitivity model; the displayed IV sensitivity weights are not weights from the primary GLMM. Navy horizontal lines are exact 95% Clopper–Pearson confidence intervals. The burgundy diamond shows the primary binomial–normal GLMM summary and its classical Wald z 95% confidence interval; the thin dashed burgundy line below it shows the 95% prediction interval. τ^2^ is reported on the logit scale.

**Figure 5:**
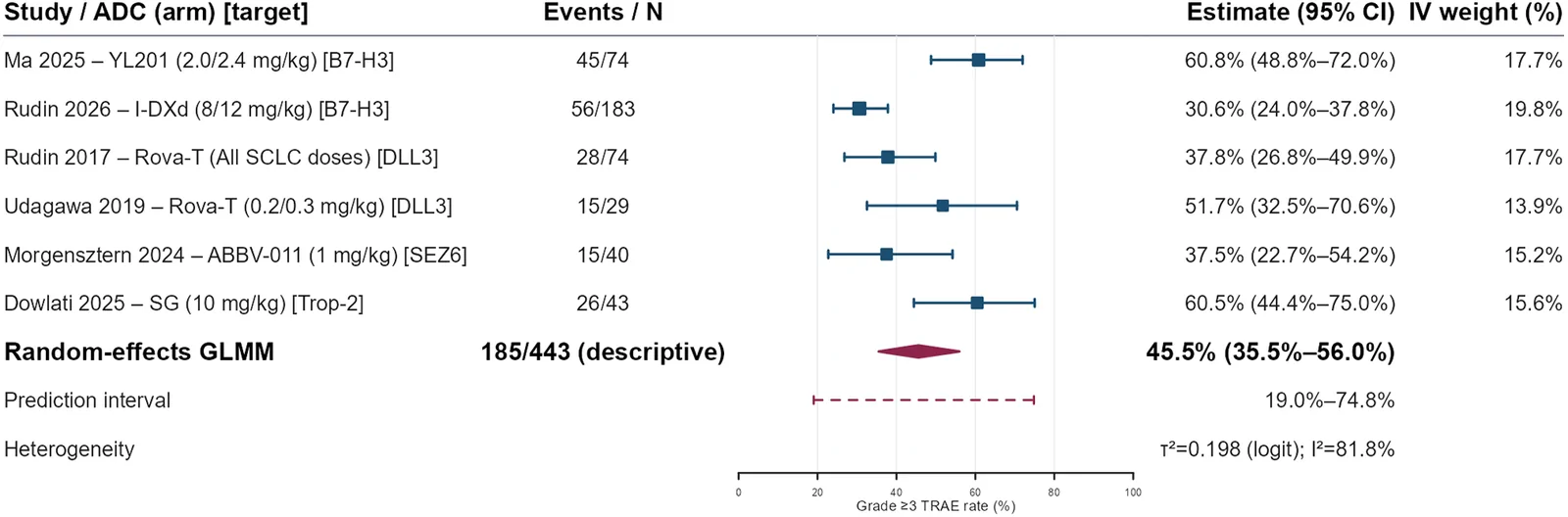
Study-level incidence of grade ≥3 treatment-related adverse events for later-line ADC monotherapy. Squares show study proportions, with area proportional to the inverse-variance logit random-effects weight from the REML sensitivity model; the displayed IV sensitivity weights are not weights from the primary GLMM. Navy horizontal lines are exact 95% Clopper–Pearson confidence intervals. The burgundy diamond shows the primary binomial–normal GLMM summary and its classical Wald z 95% confidence interval; the thin dashed burgundy line below it shows the 95% prediction interval. The TRINITY grade 3–4 count was not pooled because fatal drug-related events were reported separately and a non-overlapping grade ≥3 total was unavailable. τ^2^ is reported on the logit scale.

The TRINITY report presented grade 3–4 TRAEs in 135 of 339 patients and listed 10 grade 5 drug-related events separately. Because a non-overlapping grade ≥3 total could not be derived, the 135/339 value was retained descriptively as grade 3–4 TRAE and was excluded from the grade ≥3 TRAE meta-analysis.

Any-grade TEAEs, serious TEAEs, any-grade TRAEs, and serious TRAEs were reported by six, five, seven, and five later-line monotherapy cohorts, respectively. Their model estimates were 98.1% (95% CI, 95.9%–99.2%), 47.1% (95% CI, 39.7%–54.7%), 93.0% (95% CI, 85.0%–96.9%), and 20.5% (95% CI, 12.9%–31.0%) (**Supplementary Table S6**). The number of contributing studies and the applicable safety populations varied by outcome; these pooled proportions should therefore not be compared with one another as if they arose from a common denominator or uniform attribution process.

Common adverse events were not quantitatively pooled because reporting thresholds, preferred terms, regimen contexts, and attribution rules were inconsistent. Study-specific data were retained for descriptive reporting, but the outcome definitions were not sufficiently comparable for meta-analysis.

### 3.6 Context-specific evidence outside the primary meta-analysis

Frontline evidence consisted predominantly of ADC-containing combination regimens, including lorvotuzumab mertansine with carboplatin–etoposide and rovalpituzumab tesirine administered with or around platinum–etoposide. Later-line combination evidence included rovalpituzumab tesirine with budigalimab and with nivolumab with or without ipilimumab. MERU evaluated rovalpituzumab tesirine as post-induction maintenance. Study-specific response and safety proportions for these regimens are shown in **Supplementary Table S7** with exact CIs. They were not pooled with later-line monotherapy because chemotherapy, immunotherapy, timing of ADC exposure, and patient selection could each affect both efficacy and toxicity.

The randomized comparisons did not establish a survival advantage for the evaluated ADC-containing interventions in their respective settings. TAHOE favored topotecan rather than rovalpituzumab tesirine for OS and PFS, MERU did not show an OS benefit from rovalpituzumab tesirine maintenance versus placebo, and the frontline lorvotuzumab mertansine comparison did not establish a survival benefit (17, 23, 24). These findings were retained as comparative trial evidence and were not recast as single-arm survival proportions. Consequently, the apparently different response proportions across frontline combination, later-line monotherapy, later-line combination, and maintenance settings cannot be interpreted as treatment-line effects.

No pooled landmark PFS or OS estimate was calculated because censoring-aware uncertainty was unavailable or inconsistently reported. Kaplan–Meier probabilities and numbers at risk were not converted to ordinary event counts, and this exclusion is documented in **Supplementary Table S3**. An observed single-arm survival proportion was therefore not interpreted as evidence of survival benefit.

### 3.7 Exploratory description by molecular target

Target-stratified analyses were restricted to later-line monotherapy. Three B7-H3 studies contributed to the exploratory ORR synthesis, which yielded 50.9% (95% CI, 40.2%–61.5%; 95% prediction interval, 16.9%–84.1%); four DLL3 studies yielded 13.7% (95% CI, 11.4%–16.4%; 95% prediction interval, 10.1%–18.3%) (**Figure 6**). The corresponding exploratory DCR summaries were 87.3% (95% CI, 83.1%–90.6%) and 57.4% (95% CI, 42.3%–71.2%) (**Supplementary Table S8**). The B7-H3 estimates were based on three studies, and the DLL3 estimates combined different ADC programs and trial eras. Trop-2 (two studies) and SEZ6 (one study) were displayed as study-level estimates but were not pooled.

**Figure 6:**
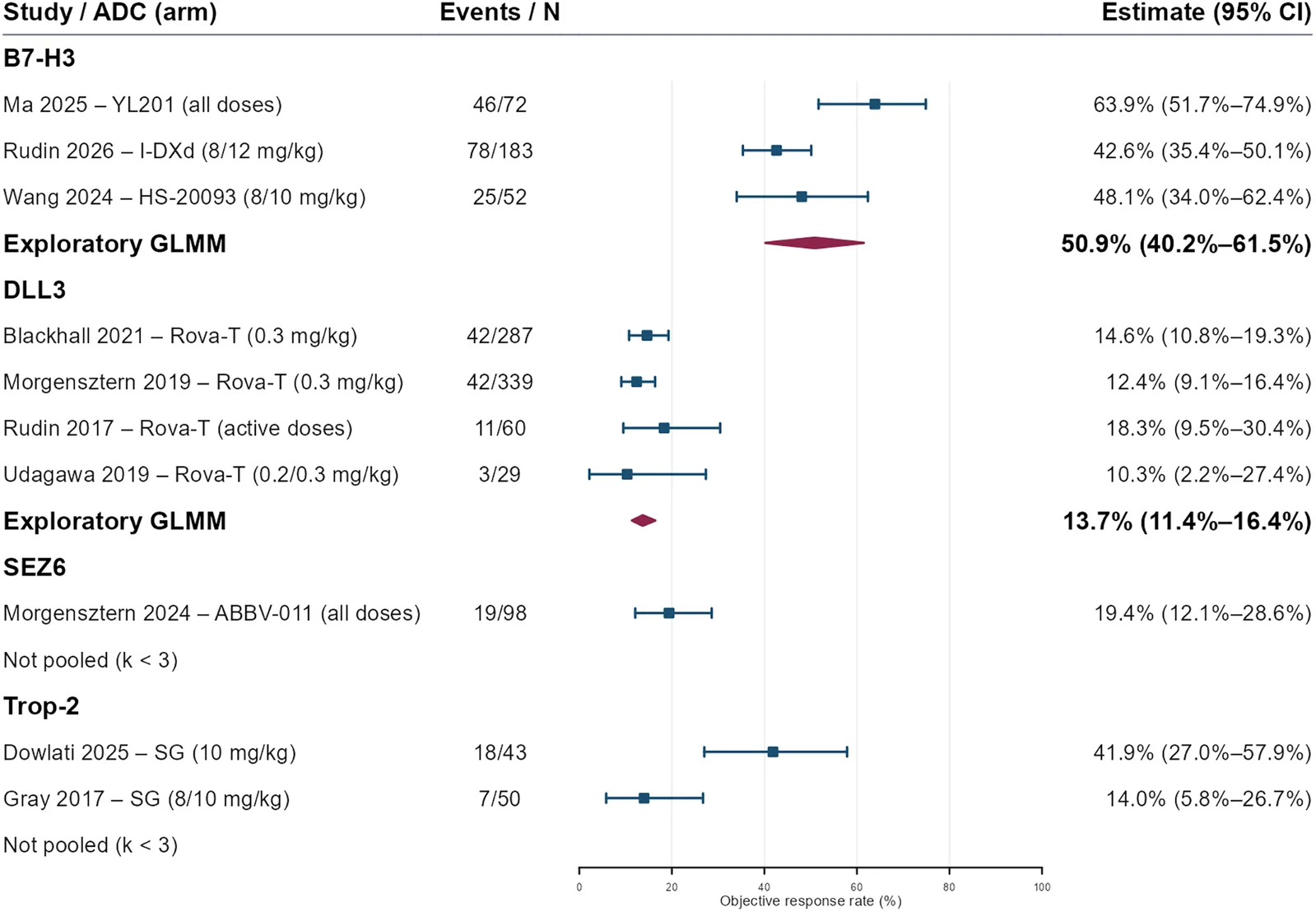
Exploratory objective response rates by molecular target among later-line ADC monotherapy studies. Study squares have a fixed size and do not encode analytical weight. Within-target GLMM summaries are shown only for B7-H3 (k=3; τ^2^=0.095; I^2^=78.1%; 95% prediction interval, 16.9%–84.1%) and DLL3 (k=4; τ^2^=0.000; I^2^=0%; 95% prediction interval, 10.1%–18.3%). These small, confounded groups provide within-target descriptive summaries only; no between-target test, indirect comparison, or ranking was performed. Trop-2 and SEZ6 were not pooled because k<3.

No between-target test or ranking was performed. Differences in the observed point estimates could reflect the ADC molecule, payload, linker, dose, eligibility criteria, prior treatment, outcome assessment, or study maturity rather than the target antigen itself (28, 29).

### 3.8 Model and influence analyses

The inverse-variance logit model with restricted maximum likelihood estimation and Hartung–Knapp inference produced closely aligned estimates for ORR (26.1%), DCR (72.3%), grade ≥3 TEAEs (62.7%), and grade ≥3 TRAEs (45.7%) relative to the primary GLMM (**Supplementary Table S6**). Leave-one-study-out estimates ranged from 22.4% to 27.9% for ORR, 69.6% to 76.2% for DCR, 62.2% to 64.0% for grade ≥3 TEAEs, and 41.8% to 49.6% for grade ≥3 TRAEs (**Supplementary Figures S2**–**S5**). A separate cohort-representation sensitivity analysis using recommended or expansion efficacy cohorts yielded ORR 26.9% (95% CI, 17.0%–39.9%) and DCR 72.9% (95% CI, 60.2%–82.7%). These analyses describe model dependence and individual-study influence. They do not overcome the clinical heterogeneity, non-comparative design, or small number of studies and are therefore not described as evidence of robustness.

### 3.9 Small-study effects

Small-study effects were not formally assessed. The number of studies was limited for most outcomes, and funnel-plot asymmetry or regression-based tests would have been difficult to interpret for heterogeneous single-arm proportions. Selective publication and selective outcome reporting therefore cannot be excluded.

## 4 Discussion

### 4.1 Principal findings

This review provides a context-specific synthesis of ADC evidence in advanced SCLC. After resolving overlapping reports, dose cohorts, analysis-set denominators, and clinical contexts, the quantitative analysis was restricted to second-line or later ADC monotherapy. Within that setting, the pooled ORR and DCR indicated measurable antitumor activity, while clinically important adverse events were frequent. The wide prediction intervals for several outcomes, particularly ORR, DCR, and grade ≥3 TRAEs, show that the average estimates conceal substantial variation across individual ADC programs. They should not be interpreted as common effects applicable to every drug, dose, or patient population.

The separation of clinical contexts materially changes the interpretation of the evidence. Frontline studies largely evaluated ADC–chemotherapy regimens, later-line combination studies added immune-checkpoint blockade, and MERU evaluated post-induction maintenance. Pooling these regimens under a single treatment-line label would attribute the effects of concomitant treatment and patient selection to the ADC itself. The descriptive differences across contexts therefore do not show that ADCs are intrinsically more active in the first-line setting. The randomized studies also did not establish a survival advantage for the evaluated ADC-containing interventions. Single-arm ORR, DCR, or landmark survival proportions cannot substitute for a controlled survival comparison.

### 4.2 Interpretation of the efficacy evidence

The later-line monotherapy ORR summarizes heterogeneous agents with different targets, linkers, payloads, doses, and stages of development. Its prediction interval is consequently more informative for generalizability than the confidence interval around the average alone. A high DCR should also be interpreted carefully because stable disease contributed substantially in several studies, and the minimum duration required for stable disease was not uniform. Response and disease-control estimates are therefore signals of antitumor activity, not evidence that the agents improve PFS or OS.

The exploratory B7-H3 estimates were numerically different from those for DLL3 in the available studies, but the data do not isolate a target effect. The B7-H3 evidence came from only three studies of contemporary ADC platforms, whereas the DLL3 group included several rovalpituzumab tesirine programs conducted across different phases, doses, and clinical settings. Differences in payload potency, linker stability, dose intensity, biomarker enrichment, prior therapy, and follow-up could all produce the observed separation. Trop-2 and SEZ6 had too few studies for pooling. These data can inform the design of prospective biomarker-stratified trials, but they do not support ranking targets or recommending a target-specific agent in clinical practice.

### 4.3 Interpretation of the safety evidence

Severe toxicity was common in the later-line monotherapy evidence base, although the magnitude differed by safety definition and contributing studies. TEAEs include events arising after treatment regardless of attribution, whereas TRAEs depend on investigator judgment; their rates are not interchangeable. In addition, safety analysis sets differed from efficacy populations in several trials, and some studies reported grade 3–4 events separately from fatal events or used drug-related rather than all-cause serious-event categories. These distinctions were retained during data extraction; outcomes that could not be harmonized reliably were not pooled.

The decision not to pool individual common adverse events reflects a limitation of the source evidence rather than an absence of clinically relevant toxicity. Trials applied different reporting thresholds, grouped preferred terms differently, and evaluated monotherapy and combination regimens with distinct background toxicities. Exact event counts are therefore more interpretable than a synthetic average that would combine different attribution rules and selective-reporting mechanisms. The available data warrant careful toxicity characterization in future trials, but they do not permit comparative claims about the safety of different targets or ADCs.

### 4.4 Relation to the existing evidence

Previous reviews have described the expanding ADC pipeline in SCLC and response activity across individual programs (30–34). In this analysis, clinical context was defined before pooling, and overlapping reports, nested dose cohorts, and outcome-specific analysis populations were reconciled. The later peer-reviewed Calvo report replaced the conference abstract, and the mixed-histology SC-002 cohort was excluded from SCLC-specific quantitative estimates. These decisions narrowed the synthesis and improved its interpretability.

Separate pooled proportions are not comparative effects. Small target subgroups cannot establish target superiority, and stability in leave-one-study-out analyses does not remove between-study heterogeneity or design bias. MINORS scores describe selected methodological features of non-randomized reports; they do not make early-phase single-arm evidence moderate or high certainty. No certainty label was assigned because GRADE was not performed.

### 4.5 Strengths and limitations

Each numerator and denominator was linked to the appropriate cohort and analysis population, and overlapping reports and dependent cohorts were resolved before pooling. The primary GLMM preserves the binomial likelihood without the inverse Freeman–Tukey transformation. It was complemented by a prespecified logit random-effects model, prediction intervals, influence analyses, and study-level forest plots that show variation hidden by summary-only figures.

Most evidence came from early-phase, non-randomized, open-label studies. The single-arm estimates are therefore sensitive to differences in patient selection, prior therapy, response assessment, and follow-up. The structured arm-level assessment identified additional concerns about analysis populations, response ascertainment, selective reporting, and dose-cohort representativeness. Because this descriptive assessment is not a validated summary instrument, it records potential sources of bias but does not provide an overall risk-of-bias score.

Clinical and statistical heterogeneity was substantial for several principal outcomes, and the small number of studies limited examination of effect modifiers. Target groups were confounded by the ADC molecule, payload, linker, dose, trial era, and patient characteristics; ecological comparisons were therefore unreliable. Safety definitions, attribution rules, and reporting thresholds were inconsistent, and some serious-event categories could not be harmonized. Censoring-aware uncertainty for landmark survival was not consistently available; a valid pooled survival analysis was therefore not possible. Selective publication and outcome reporting remain possible because formal asymmetry assessment was uninformative in this setting. No outcome-level GRADE assessment was performed, and the methodological-quality ratings should not be interpreted as certainty of clinical benefit.

### 4.6 Implications for research

Current evidence supports further trial development, not practice recommendations. Randomized studies should be tailored to clinical context and distinguish monotherapy, combination therapy, and maintenance. They should use harmonized definitions for response, disease control, PFS, OS, patient-reported outcomes, and toxicity, and should report Kaplan–Meier estimates with confidence intervals or other censoring-aware measures. Safety reports should distinguish TEAEs from TRAEs and state whether grade ≥3 categories include fatal events.

Target expression should be evaluated prospectively using prespecified assays and thresholds, with treatment-by-biomarker interaction analyses where sample size permits. Comparative trials are needed to determine whether an apparent signal is attributable to the target antigen, the ADC platform, or differences in the study population.

## 5 Conclusions

Later-line ADC monotherapy showed response activity in advanced SCLC, but clinically important toxicity was frequent and several outcomes varied substantially between studies. Evidence from frontline combinations, later-line combinations, and maintenance is not exchangeable with monotherapy data. The available single-arm evidence establishes neither survival benefit nor comparative superiority by agent or target. Exploratory target signals should inform prospective study design rather than treatment selection. Randomized, context-specific trials with standardized outcome and safety reporting are needed to define the clinical role of ADCs in SCLC.

## Data Availability

The original contributions presented in the study are included in the article/**Supplementary Material.** Further inquiries can be directed to the corresponding author.
