# Supplementary material for: Antibody–drug conjugates in advanced small-cell lung cancer: a systematic review and single-arm meta-analysis of later-line monotherapy
Source: Front Immunol. 2026 Oct 7;17:1929911. doi: 10.3389/fimmu.2026.1929911 (PMC13683592; doi:10.3389/fimmu.2026.1929911)
Supplement: Supplementary file 1 [file Table1.docx]

Supplementary materials

Antibody–Drug Conjugates in Advanced Small-Cell Lung Cancer: A Systematic Review and Single-Arm Meta-Analysis of Later-Line Monotherapy

Xinmiao Chen, Shaoxun Li and Huiying Li

**Review registration: PROSPERO CRD420261354772.**

**Table S1A. Complete search strategies, search dates, and stepwise retrieval counts for bibliographic databases and trial registries.**

The bibliographic database searches were updated in 28 August 2026. No randomized controlled trial filter was applied. The PubMed target-enriched strategy was used as a sensitivity search.

**Bibliographic databases**

| **PubMed** | **Platform: PubMed** | **Search date: 28 August 2026; Final retrieval: 1,595** |
| --- | --- | --- |
| **Search no.** | **Search strategy** | **Results** |
| #1 | "Carcinoma, Small Cell Lung"[Mesh] OR "small cell lung cancer"[Title/Abstract] OR "small-cell lung cancer"[Title/Abstract] OR "small cell lung carcinoma"[Title/Abstract] OR "small-cell lung carcinoma"[Title/Abstract] OR "small cell carcinoma of the lung"[Title/Abstract] OR "carcinoma, small cell lung"[Title/Abstract] OR "oat cell lung cancer"[Title/Abstract] OR "oat cell carcinoma of lung"[Title/Abstract] OR SCLC[Title/Abstract] | 123,401 |
| #2 | "Immunoconjugates"[Mesh] OR "antibody-drug conjugate"[Title/Abstract] OR "antibody-drug conjugates"[Title/Abstract] OR "antibody drug conjugate"[Title/Abstract] OR "antibody drug conjugates"[Title/Abstract] OR immunoconjugate*[Title/Abstract] OR "antibody conjugate"[Title/Abstract] OR "antibody conjugates"[Title/Abstract] OR ADC[Title/Abstract] OR ADCs[Title/Abstract] | 45,556 |
| #3 | "rovalpituzumab tesirine"[Title/Abstract] OR rovalpituzumab[Title/Abstract] OR "Rova-T"[Title/Abstract] OR "sacituzumab govitecan"[Title/Abstract] OR sacituzumab[Title/Abstract] OR "IMMU-132"[Title/Abstract] OR "ifinatamab deruxtecan"[Title/Abstract] OR ifinatamab[Title/Abstract] OR "DS-7300"[Title/Abstract] OR "DS-7300a"[Title/Abstract] OR "HS-20093"[Title/Abstract] OR "ABBV-011"[Title/Abstract] OR "lorvotuzumab mertansine"[Title/Abstract] OR lorvotuzumab[Title/Abstract] OR "IMGN901"[Title/Abstract] OR "SC-002"[Title/Abstract] OR "YL201"[Title/Abstract] | 956 |
| #4 | DLL3[Title/Abstract] OR "delta-like ligand 3"[Title/Abstract] OR "delta like ligand 3"[Title/Abstract] OR "B7-H3"[Title/Abstract] OR B7H3[Title/Abstract] OR CD276[Title/Abstract] OR "Trop-2"[Title/Abstract] OR Trop2[Title/Abstract] OR TACSTD2[Title/Abstract] OR SEZ6[Title/Abstract] OR CD56[Title/Abstract] OR NCAM1[Title/Abstract] | 17,205 |
| #5 | #2 OR #3 | 46,512 |
| #6 | #1 AND (#2 OR #3 OR #4) [sensitivity search] | 2,145 |
| #7 | #1 AND (#2 OR #3) [main search] | 1,595 |

| **Embase** | **Platform: Embase.com** | **Search date: 28 August 2026 ; Final retrieval: 3,545** |
| --- | --- | --- |
| **Search no.** | **Search strategy** | **Results** |
| #1 | 'small cell lung cancer'/exp OR 'small cell lung cancer':ti,ab,kw OR 'small-cell lung cancer':ti,ab,kw OR 'small cell lung carcinoma':ti,ab,kw OR 'small-cell lung carcinoma':ti,ab,kw OR 'small cell carcinoma of the lung':ti,ab,kw OR 'carcinoma, small cell lung':ti,ab,kw OR 'oat cell lung cancer':ti,ab,kw OR 'oat cell carcinoma of lung':ti,ab,kw OR sclc:ti,ab,kw | 217,764 |
| #2 | 'antibody conjugate'/exp OR 'antibody-drug conjugate':ti,ab,kw OR 'antibody-drug conjugates':ti,ab,kw OR 'antibody drug conjugate':ti,ab,kw OR 'antibody drug conjugates':ti,ab,kw OR immunoconjugate*:ti,ab,kw OR 'antibody conjugate':ti,ab,kw OR 'antibody conjugates':ti,ab,kw OR adc:ti,ab,kw OR adcs:ti,ab,kw | 66,106 |
| #3 | 'rovalpituzumab tesirine':ti,ab,kw OR rovalpituzumab:ti,ab,kw OR 'rova-t':ti,ab,kw OR 'sacituzumab govitecan':ti,ab,kw OR sacituzumab:ti,ab,kw OR 'immu-132':ti,ab,kw OR 'ifinatamab deruxtecan':ti,ab,kw OR ifinatamab:ti,ab,kw OR 'ds-7300':ti,ab,kw OR 'ds-7300a':ti,ab,kw OR 'hs-20093':ti,ab,kw OR 'abbv-011':ti,ab,kw OR 'lorvotuzumab mertansine':ti,ab,kw OR lorvotuzumab:ti,ab,kw OR imgn901:ti,ab,kw OR 'sc-002':ti,ab,kw OR yl201:ti,ab,kw | 2,635 |
| #4 | dll3:ti,ab,kw OR 'delta-like ligand 3':ti,ab,kw OR 'delta like ligand 3':ti,ab,kw OR 'b7-h3':ti,ab,kw OR b7h3:ti,ab,kw OR cd276:ti,ab,kw OR 'trop-2':ti,ab,kw OR trop2:ti,ab,kw OR tacstd2:ti,ab,kw OR sez6:ti,ab,kw OR cd56:ti,ab,kw OR ncam1:ti,ab,kw | 33,062 |
| #5 | #2 OR #3 | 66,834 |
| #6 | #1 AND #5 [main search] | 3,545 |

| **Cochrane Library** | **Platform: Search Manager, Trials** | **Search date: 28 August 2026; Final retrieval: 275** |
| --- | --- | --- |
| **Search no.** | **Search strategy** | **Results** |
| #1 | MeSH descriptor: [Small Cell Lung Carcinoma] explode all trees | 739 |
| #2 | ("small cell lung cancer" or "small-cell lung cancer" or "small cell lung carcinoma" or "small-cell lung carcinoma" or "small cell carcinoma of the lung" or "carcinoma, small cell lung" or "oat cell lung cancer" or "oat cell carcinoma of lung" or SCLC):ti,ab,kw | 20,894 |
| #3 | #1 OR #2 | 20,894 |
| #4 | MeSH descriptor: [Immunoconjugates] explode all trees | 792 |
| #5 | ("antibody-drug conjugate" or "antibody-drug conjugates" or "antibody drug conjugate" or "antibody drug conjugates" or immunoconjugate or immunoconjugates or "antibody conjugate" or "antibody conjugates" or ADC or ADCs):ti,ab,kw | 3,306 |
| #6 | #4 OR #5 | 3,651 |
| #7 | ("rovalpituzumab tesirine" or rovalpituzumab or "Rova-T" or "sacituzumab govitecan" or sacituzumab or "IMMU-132" or "ifinatamab deruxtecan" or ifinatamab or "DS-7300" or "DS-7300a" or "HS-20093" or "ABBV-011" or "lorvotuzumab mertansine" or lorvotuzumab or IMGN901 or "SC-002" or YL201):ti,ab,kw | 409 |
| #8 | #6 OR #7 | 3,819 |
| #9 | #3 AND #8 in Trials [main search] | 275 |

| **Web of Science Core Collection** | **Platform: Web of Science Core Collection** | **Search date: 28 August 2026 ; Final retrieval: 1,778** |
| --- | --- | --- |
| **Search no.** | **Search strategy** | **Results** |
| #1 | TS=("small cell lung cancer" OR "small-cell lung cancer" OR "small cell lung carcinoma" OR "small-cell lung carcinoma" OR "small cell carcinoma of the lung" OR "carcinoma, small cell lung" OR "oat cell lung cancer" OR "oat cell carcinoma of lung" OR SCLC) | Not retained in the search log |
| #2 | TS=("antibody-drug conjugate*" OR "antibody drug conjugate*" OR immunoconjugate* OR "antibody conjugate*" OR ADC OR ADCs) | 77,842 |
| #3 | TS=("rovalpituzumab tesirine" OR rovalpituzumab OR "Rova-T" OR "sacituzumab govitecan" OR sacituzumab OR "IMMU-132" OR "ifinatamab deruxtecan" OR ifinatamab OR "DS-7300" OR "DS-7300a" OR "HS-20093" OR "ABBV-011" OR "lorvotuzumab mertansine" OR lorvotuzumab OR IMGN901 OR "SC-002" OR YL201) | 2,212 |
| #4 | #2 OR #3 | 78,807 |
| #5 | #1 AND #4 [main search] | 1,778 |

**Trial registries**

| **ClinicalTrials.gov** | **Platform: ClinicalTrials.gov** | **Search date: 28 August 2026** |
| --- | --- | --- |
| **Search no.** | **Search strategy** | **Results** |
| #1 | AREA[ConditionSearch](EXPANSION[Term]SCLC OR (EXPANSION[Term]"small cell lung cancer" AND NOT EXPANSION[Term]"non-small cell lung cancer") OR (EXPANSION[Term]"small cell lung carcinoma" AND NOT EXPANSION[Term]"non-small cell lung carcinoma") OR EXPANSION[Term]"small cell carcinoma of the lung" OR EXPANSION[Term]"carcinoma, small cell lung" OR EXPANSION[Term]"oat cell lung cancer" OR EXPANSION[Term]"oat cell carcinoma of lung") | 1,520 records |
| #2 | (AREA[InterventionSearch](EXPANSION[Term]"antibody-drug conjugate" OR EXPANSION[Term]"antibody drug conjugate" OR EXPANSION[Term]immunoconjugate OR EXPANSION[Term]"antibody conjugate")) OR (AREA[InterventionNameSearch](EXPANSION[Term]"rovalpituzumab tesirine" OR EXPANSION[Term]rovalpituzumab OR EXPANSION[Term]"Rova-T" OR EXPANSION[Term]"SC16LD6.5" OR EXPANSION[Term]"sacituzumab govitecan" OR EXPANSION[Term]sacituzumab OR EXPANSION[Term]"IMMU-132" OR EXPANSION[Term]"ifinatamab deruxtecan" OR EXPANSION[Term]ifinatamab OR EXPANSION[Term]"I-DXd" OR EXPANSION[Term]"DS-7300" OR EXPANSION[Term]"DS-7300a" OR EXPANSION[Term]"HS-20093" OR EXPANSION[Term]"ABBV-011" OR EXPANSION[Term]"lorvotuzumab mertansine" OR EXPANSION[Term]lorvotuzumab OR EXPANSION[Term]"IMGN901" OR EXPANSION[Term]"BB-10901" OR EXPANSION[Term]"huN901-DM1" OR EXPANSION[Term]"SC-002" OR EXPANSION[Term]"YL201")) | 962 records |
| #3 | (#1) AND (#2) | 46 records |

| **WHO ICTRP** | **Platform: ICTRP Advanced Search** | **Search date: 28 August 2026** |
| --- | --- | --- |
| **Search no.** | **Search strategy** | **Results** |
| #1 | Condition field: "small cell lung cancer" OR "small-cell lung cancer" OR "small cell lung carcinoma" OR "small-cell lung carcinoma" OR "small cell carcinoma of the lung" OR "oat cell lung cancer" OR "oat cell carcinoma of lung" OR SCLC | 3,761 records; 3,115 trial groups |
| #2 | Intervention field: "antibody-drug conjugate" OR "antibody-drug conjugates" OR "antibody drug conjugate" OR "antibody drug conjugates" OR immunoconjugate* OR "rovalpituzumab tesirine" OR rovalpituzumab OR "Rova-T" OR "sacituzumab govitecan" OR sacituzumab OR "IMMU-132" OR "ifinatamab deruxtecan" OR ifinatamab OR "DS-7300" OR "DS-7300a" OR "HS-20093" OR "ABBV-011" OR "lorvotuzumab mertansine" OR lorvotuzumab OR IMGN901 OR "SC-002" OR YL201 | 229 records; 161 trial groups |
| #3 | #1 AND #2 | 39 records; 22 trial groups |

Table S1B. Supplementary searches of conference abstract sources and reference lists.

| **Information source** | **Search date** | **Coverage** | **Keywords/strategy** | **Potentially relevant reports identified, n** |
| --- | --- | --- | --- | --- |
| ASCO Meeting Library | 28 August 2026 | 2015–2026 | Presentations; ("small cell lung cancer" OR SCLC) combined with ADC/class and prespecified agent/synonym terms; restricted to 2015–2026 and deduplicated by ASCO presentation ID. | 17 |
| WCLC abstracts (IASLC) | 28 August 2026 | 2015–2026 | SCLC terms AND ADC/class or named-agent terms in titles, abstracts, or keywords; all presentation types; source-level record deduplication. | 18 |
| ESMO Congress / ECC 2015 | 28 August 2026 | 2015–2026 | SCLC terms AND ADC/class or prespecified agent/synonym terms; serial title/abstract searches verified against official congress resources and supplements. | 15 |
| Reference-list searching | 28 August 2026 | Included studies and relevant reviews | Backward and forward citation searching | 7 |

Note: The ASCO searches retrieved 47 unique platform records within the date range before relevance screening; WCLC and ESMO did not provide stable cross-year raw-result totals. ECC 2015 was included as an ESMO-partner congress. Counts are not additive because the same report or study may appear in more than one source. After cross-source deduplication and eligibility assessment, no additional eligible studies were identified. ADC, antibody–drug conjugate; ASCO, American Society of Clinical Oncology; ECC, European Cancer Congress; ESMO, European Society for Medical Oncology; IASLC, International Association for the Study of Lung Cancer; SCLC, small-cell lung cancer; WCLC, World Conference on Lung Cancer.

**Table S2A. Arm- and outcome-level data used in the quantitative synthesis: molecular target, cohort, and outcome fields.**

| Record | Study/report | Trial | Clinical context | Regimen | Molecular target | Dose | Outcome | Events/N | Analysis set | Primary pool |
| --- | --- | --- | --- | --- | --- | --- | --- | --- | --- | --- |
| S2-001 | Ma et al. 2025 | NCT05434234/NCT06057922 | Later-line monotherapy | YL201 | B7-H3 | All studied doses | ORR | 46/72 | Efficacy analysis set | Yes |
| S2-002 | Ma et al. 2025 | NCT05434234/NCT06057922 | Later-line monotherapy | YL201 | B7-H3 | All studied doses | DCR | 66/72 | Efficacy analysis set | Yes |
| S2-003 | Ma et al. 2025 | NCT05434234/NCT06057922 | Later-line monotherapy | YL201 | B7-H3 | 2.0 or 2.4 mg/kg | All-grade TRAE | 73/74 | Recommended-dose SCLC safety set | Yes |
| S2-004 | Ma et al. 2025 | NCT05434234/NCT06057922 | Later-line monotherapy | YL201 | B7-H3 | 2.0 or 2.4 mg/kg | Grade ≥3 TRAE | 45/74 | Recommended-dose SCLC safety set | Yes |
| S2-005 | Ma et al. 2025 | NCT05434234/NCT06057922 | Later-line monotherapy | YL201 | B7-H3 | 2.0 or 2.4 mg/kg | Serious TRAE | 26/74 | Recommended-dose SCLC safety set | Yes |
| S2-006 | Dowlati et al. 2025 | NCT03964727 | Later-line monotherapy | Sacituzumab govitecan | Trop-2 | 10 mg/kg | ORR | 18/43 | Efficacy analysis set | Yes |
| S2-007 | Dowlati et al. 2025 | NCT03964727 | Later-line monotherapy | Sacituzumab govitecan | Trop-2 | 10 mg/kg | DCR | 36/43 | Efficacy analysis set | Yes |
| S2-008 | Dowlati et al. 2025 | NCT03964727 | Later-line monotherapy | Sacituzumab govitecan | Trop-2 | 10 mg/kg | All-grade TEAE | 43/43 | Safety analysis set | Yes |
| S2-009 | Dowlati et al. 2025 | NCT03964727 | Later-line monotherapy | Sacituzumab govitecan | Trop-2 | 10 mg/kg | Grade ≥3 TEAE | 32/43 | Safety analysis set | Yes |
| S2-010 | Dowlati et al. 2025 | NCT03964727 | Later-line monotherapy | Sacituzumab govitecan | Trop-2 | 10 mg/kg | Serious TEAE | 22/43 | Safety analysis set | Yes |
| S2-011 | Dowlati et al. 2025 | NCT03964727 | Later-line monotherapy | Sacituzumab govitecan | Trop-2 | 10 mg/kg | All-grade TRAE | 42/43 | Safety analysis set | Yes |
| S2-012 | Dowlati et al. 2025 | NCT03964727 | Later-line monotherapy | Sacituzumab govitecan | Trop-2 | 10 mg/kg | Grade ≥3 TRAE | 26/43 | Safety analysis set | Yes |
| S2-013 | Rudin et al. 2026 | NCT05280470 | Later-line monotherapy | Ifinatamab deruxtecan | B7-H3 | 8 or 12 mg/kg | ORR | 78/183 | Combined disjoint efficacy cohorts | Yes |
| S2-014 | Rudin et al. 2026 | NCT05280470 | Later-line monotherapy | Ifinatamab deruxtecan | B7-H3 | 8 or 12 mg/kg | DCR | 157/183 | Combined disjoint efficacy cohorts | Yes |
| S2-015 | Rudin et al. 2026 | NCT05280470 | Later-line monotherapy | Ifinatamab deruxtecan | B7-H3 | 8 or 12 mg/kg | All-grade TEAE | 180/183 | Combined disjoint safety cohorts | Yes |
| S2-016 | Rudin et al. 2026 | NCT05280470 | Later-line monotherapy | Ifinatamab deruxtecan | B7-H3 | 8 or 12 mg/kg | Grade ≥3 TEAE | 106/183 | Combined disjoint safety cohorts | Yes |
| S2-017 | Rudin et al. 2026 | NCT05280470 | Later-line monotherapy | Ifinatamab deruxtecan | B7-H3 | 8 or 12 mg/kg | Serious TEAE | 69/183 | Combined disjoint safety cohorts | Yes |
| S2-018 | Rudin et al. 2026 | NCT05280470 | Later-line monotherapy | Ifinatamab deruxtecan | B7-H3 | 8 or 12 mg/kg | All-grade TRAE | 158/183 | Combined disjoint safety cohorts | Yes |
| S2-019 | Rudin et al. 2026 | NCT05280470 | Later-line monotherapy | Ifinatamab deruxtecan | B7-H3 | 8 or 12 mg/kg | Grade ≥3 TRAE | 56/183 | Combined disjoint safety cohorts | Yes |
| S2-020 | Rudin et al. 2026 | NCT05280470 | Later-line monotherapy | Ifinatamab deruxtecan | B7-H3 | 8 or 12 mg/kg | Serious TRAE | 28/183 | Combined disjoint safety cohorts | Yes |
| S2-021 | Wang et al. 2024 | NCT05276609 | Later-line monotherapy | HS-20093 | B7-H3 | 8 or 10 mg/kg | ORR | 25/52 | Combined disjoint dose cohorts | Yes |
| S2-022 | Wang et al. 2024 | NCT05276609 | Later-line monotherapy | HS-20093 | B7-H3 | 8 or 10 mg/kg | DCR | 45/52 | Combined disjoint dose cohorts | Yes |
| S2-023 | Morgensztern et al. 2024 | NCT03639194 | Later-line monotherapy | ABBV-011 | SEZ6 | All studied doses | ORR | 19/98 | Overall efficacy-evaluable set | Yes |
| S2-024 | Morgensztern et al. 2024 | NCT03639194 | Later-line monotherapy | ABBV-011 | SEZ6 | All studied doses | DCR | 68/98 | Overall efficacy-evaluable set | Yes |
| S2-025 | Morgensztern et al. 2024 | NCT03639194 | Later-line monotherapy | ABBV-011 | SEZ6 | 1 mg/kg | All-grade TEAE | 39/40 | RP2D safety cohort | Yes |
| S2-026 | Morgensztern et al. 2024 | NCT03639194 | Later-line monotherapy | ABBV-011 | SEZ6 | 1 mg/kg | Grade ≥3 TEAE | 26/40 | RP2D safety cohort | Yes |
| S2-027 | Morgensztern et al. 2024 | NCT03639194 | Later-line monotherapy | ABBV-011 | SEZ6 | 1 mg/kg | Serious TEAE | 18/40 | RP2D safety cohort | Yes |
| S2-028 | Morgensztern et al. 2024 | NCT03639194 | Later-line monotherapy | ABBV-011 | SEZ6 | 1 mg/kg | All-grade TRAE | 31/40 | RP2D safety cohort | Yes |
| S2-029 | Morgensztern et al. 2024 | NCT03639194 | Later-line monotherapy | ABBV-011 | SEZ6 | 1 mg/kg | Grade ≥3 TRAE | 15/40 | RP2D safety cohort | Yes |
| S2-030 | Morgensztern et al. 2024 | NCT03639194 | Later-line monotherapy | ABBV-011 | SEZ6 | 1 mg/kg | Serious TRAE | 3/40 | RP2D safety cohort | Yes |
| S2-031 | Blackhall et al. 2021 | NCT03061812 | Later-line monotherapy | Rovalpituzumab tesirine | DLL3 | 0.3 mg/kg | ORR | 42/287 | Rova-T efficacy set | Yes |
| S2-032 | Blackhall et al. 2021 | NCT03061812 | Later-line monotherapy | Rovalpituzumab tesirine | DLL3 | 0.3 mg/kg | DCR | 103/287 | Rova-T efficacy set | Yes |
| S2-033 | Blackhall et al. 2021 | NCT03061812 | Later-line monotherapy | Rovalpituzumab tesirine | DLL3 | 0.3 mg/kg | All-grade TEAE | 273/287 | Rova-T safety set | Yes |
| S2-034 | Blackhall et al. 2021 | NCT03061812 | Later-line monotherapy | Rovalpituzumab tesirine | DLL3 | 0.3 mg/kg | Grade ≥3 TEAE | 183/287 | Rova-T safety set | Yes |
| S2-035 | Blackhall et al. 2021 | NCT03061812 | Later-line monotherapy | Rovalpituzumab tesirine | DLL3 | 0.3 mg/kg | Serious TEAE | 160/287 | Rova-T safety set | Yes |
| S2-036 | Morgensztern et al. 2020 | NCT02500914 | Later-line mixed histology | SC-002 | DLL3 | 0.025-0.4 mg/kg | ORR | 5/35 | Combined SCLC/LCNEC efficacy set | No |
| S2-037 | Morgensztern et al. 2020 | NCT02500914 | Later-line mixed histology | SC-002 | DLL3 | 0.025-0.4 mg/kg | DCR | 19/35 | Combined SCLC/LCNEC efficacy set | No |
| S2-038 | Morgensztern et al. 2020 | NCT02500914 | Later-line mixed histology | SC-002 | DLL3 | 0.025-0.4 mg/kg | All-grade TEAE | 35/35 | Combined SCLC/LCNEC safety set | No |
| S2-039 | Morgensztern et al. 2020 | NCT02500914 | Later-line mixed histology | SC-002 | DLL3 | 0.025-0.4 mg/kg | Grade ≥3 TEAE | 24/35 | Combined SCLC/LCNEC safety set | No |
| S2-040 | Morgensztern et al. 2020 | NCT02500914 | Later-line mixed histology | SC-002 | DLL3 | 0.025-0.4 mg/kg | Serious TEAE | 23/35 | Combined SCLC/LCNEC safety set | No |
| S2-041 | Morgensztern et al. 2020 | NCT02500914 | Later-line mixed histology | SC-002 | DLL3 | 0.025-0.4 mg/kg | All-grade TRAE | 28/35 | Combined SCLC/LCNEC safety set | No |
| S2-042 | Morgensztern et al. 2020 | NCT02500914 | Later-line mixed histology | SC-002 | DLL3 | 0.025-0.4 mg/kg | Serious TRAE | 13/35 | Combined SCLC/LCNEC safety set | No |
| S2-043 | Morgensztern et al. 2019 | NCT02674568 | Later-line monotherapy | Rovalpituzumab tesirine | DLL3 | 0.3 mg/kg | ORR | 42/339 | Treated efficacy set | Yes |
| S2-044 | Morgensztern et al. 2019 | NCT02674568 | Later-line monotherapy | Rovalpituzumab tesirine | DLL3 | 0.3 mg/kg | DCR | 236/339 | Treated efficacy set | Yes |
| S2-045 | Morgensztern et al. 2019 | NCT02674568 | Later-line monotherapy | Rovalpituzumab tesirine | DLL3 | 0.3 mg/kg | All-grade TEAE | 335/339 | Safety set | Yes |
| S2-046 | Morgensztern et al. 2019 | NCT02674568 | Later-line monotherapy | Rovalpituzumab tesirine | DLL3 | 0.3 mg/kg | Grade ≥3 TEAE | 213/339 | Safety set | Yes |
| S2-047 | Morgensztern et al. 2019 | NCT02674568 | Later-line monotherapy | Rovalpituzumab tesirine | DLL3 | 0.3 mg/kg | Serious TRAE | 100/339 | Safety set | Yes |
| S2-048 | Morgensztern et al. 2019 | NCT02674568 | Later-line monotherapy | Rovalpituzumab tesirine | DLL3 | 0.3 mg/kg | All-grade TRAE | 308/339 | Safety set | Yes |
| S2-049 | Morgensztern et al. 2019 | NCT02674568 | Later-line monotherapy | Rovalpituzumab tesirine | DLL3 | 0.3 mg/kg | grade 3–4 TRAE | 135/339 | Safety set | No |
| S2-050 | Calvo et al. 2021 | NCT03000257 | Later-line combination | Rovalpituzumab tesirine + budigalimab | DLL3 | Reported regimen | ORR | 7/29 | Efficacy-evaluable set | No |
| S2-051 | Calvo et al. 2021 | NCT03000257 | Later-line combination | Rovalpituzumab tesirine + budigalimab | DLL3 | Reported regimen | DCR | 22/29 | Efficacy-evaluable set | No |
| S2-052 | Calvo et al. 2021 | NCT03000257 | Later-line combination | Rovalpituzumab tesirine + budigalimab | DLL3 | Reported regimen | All-grade TEAE | 31/31 | Safety set | No |
| S2-053 | Calvo et al. 2021 | NCT03000257 | Later-line combination | Rovalpituzumab tesirine + budigalimab | DLL3 | Reported regimen | Grade ≥3 TEAE | 26/31 | Safety set | No |
| S2-054 | Calvo et al. 2021 | NCT03000257 | Later-line combination | Rovalpituzumab tesirine + budigalimab | DLL3 | Reported regimen | All-grade TRAE | 26/31 | Safety set | No |
| S2-055 | Calvo et al. 2021 | NCT03000257 | Later-line combination | Rovalpituzumab tesirine + budigalimab | DLL3 | Reported regimen | Grade ≥3 TRAE | 17/31 | Safety set | No |
| S2-056 | Rudin et al. 2017 | NCT01901653 | Later-line monotherapy | Rovalpituzumab tesirine | DLL3 | Active-dose schedules | ORR | 11/60 | Active-dose response-evaluable set | Yes |
| S2-057 | Rudin et al. 2017 | NCT01901653 | Later-line monotherapy | Rovalpituzumab tesirine | DLL3 | Active-dose schedules | DCR | 41/60 | Active-dose response-evaluable set | Yes |
| S2-058 | Rudin et al. 2017 | NCT01901653 | Later-line monotherapy | Rovalpituzumab tesirine | DLL3 | All SCLC doses | All-grade TRAE | 65/74 | SCLC safety set | Yes |
| S2-059 | Rudin et al. 2017 | NCT01901653 | Later-line monotherapy | Rovalpituzumab tesirine | DLL3 | All SCLC doses | Grade ≥3 TRAE | 28/74 | SCLC safety set | Yes |
| S2-060 | Rudin et al. 2017 | NCT01901653 | Later-line monotherapy | Rovalpituzumab tesirine | DLL3 | All SCLC doses | Serious TRAE | 28/74 | SCLC safety set | No |
| S2-061 | Gray et al. 2017 | NCT01631552 | Later-line monotherapy | Sacituzumab govitecan | Trop-2 | 8 or 10 mg/kg | ORR | 7/50 | ITT efficacy set | Yes |
| S2-062 | Gray et al. 2017 | NCT01631552 | Later-line monotherapy | Sacituzumab govitecan | Trop-2 | 8 or 10 mg/kg | DCR | 28/50 | ITT efficacy set | Yes |
| S2-063 | Johnson et al. 2021 | NCT03033511 | Post-induction maintenance | Rovalpituzumab tesirine | DLL3 | 0.3 mg/kg | ORR | 28/318 | Response-evaluable Rova-T arm | No |
| S2-064 | Johnson et al. 2021 | NCT03033511 | Post-induction maintenance | Rovalpituzumab tesirine | DLL3 | 0.3 mg/kg | DCR | 211/318 | Response-evaluable Rova-T arm | No |
| S2-065 | Johnson et al. 2021 | NCT03033511 | Post-induction maintenance | Rovalpituzumab tesirine | DLL3 | 0.3 mg/kg | All-grade TEAE | 343/368 | Rova-T safety set | No |
| S2-066 | Johnson et al. 2021 | NCT03033511 | Post-induction maintenance | Rovalpituzumab tesirine | DLL3 | 0.3 mg/kg | Grade ≥3 TEAE | 217/368 | Rova-T safety set | No |
| S2-067 | Johnson et al. 2021 | NCT03033511 | Post-induction maintenance | Rovalpituzumab tesirine | DLL3 | 0.3 mg/kg | Serious TEAE | 157/368 | Rova-T safety set | No |
| S2-068 | Johnson et al. 2021 | NCT03033511 | Post-induction maintenance | Rovalpituzumab tesirine | DLL3 | 0.3 mg/kg | All-grade TRAE | 290/368 | Rova-T safety set | No |
| S2-069 | Socinski et al. 2017 | NCT01237678 | Frontline combination | Lorvotuzumab mertansine + carboplatin/etoposide | CD56 | Reported regimen | ORR | 55/82 | Response-evaluable experimental arm | No |
| S2-070 | Socinski et al. 2017 | NCT01237678 | Frontline combination | Lorvotuzumab mertansine + carboplatin/etoposide | CD56 | Reported regimen | DCR | 75/82 | Response-evaluable experimental arm | No |
| S2-071 | Socinski et al. 2017 | NCT01237678 | Frontline combination | Lorvotuzumab mertansine + carboplatin/etoposide | CD56 | Reported regimen | All-grade TEAE | 94/94 | Experimental-arm safety set | No |
| S2-072 | Socinski et al. 2017 | NCT01237678 | Frontline combination | Lorvotuzumab mertansine + carboplatin/etoposide | CD56 | Reported regimen | Grade ≥3 TEAE | 92/94 | Experimental-arm safety set | No |
| S2-073 | Socinski et al. 2017 | NCT01237678 | Frontline combination | Lorvotuzumab mertansine + carboplatin/etoposide | CD56 | Reported regimen | Serious TEAE | 54/94 | Experimental-arm safety set | No |

Table S2A (continued).

| Record | Study/report | Trial | Clinical context | Regimen | Molecular target | Dose | Outcome | Events/N | Analysis set | Primary pool |
| --- | --- | --- | --- | --- | --- | --- | --- | --- | --- | --- |
| S2-074 | Hann et al. 2021 | NCT02819999 | Frontline combination | Rovalpituzumab tesirine + concurrent platinum/etoposide | DLL3 | 0.1 or 0.2 mg/kg | ORR | 7/14 | Cohort 3 | No |
| S2-075 | Hann et al. 2021 | NCT02819999 | Frontline combination | Rovalpituzumab tesirine + concurrent platinum/etoposide | DLL3 | 0.1 or 0.2 mg/kg | All-grade TEAE | 14/14 | Cohort 3 | No |
| S2-076 | Hann et al. 2021 | NCT02819999 | Frontline combination | Rovalpituzumab tesirine + concurrent platinum/etoposide | DLL3 | 0.1 or 0.2 mg/kg | Grade ≥3 TEAE | 13/14 | Cohort 3 | No |
| S2-077 | Hann et al. 2021 | NCT02819999 | Frontline combination | Rovalpituzumab tesirine + concurrent platinum/etoposide | DLL3 | 0.1 or 0.2 mg/kg | Serious TEAE | 6/14 | Cohort 3 | No |
| S2-078 | Malhotra et al. 2021 | NCT03026166 | Later-line combination | Rovalpituzumab tesirine + nivolumab +/- ipilimumab | DLL3 | Reported regimens | ORR | 12/40 | Combined efficacy-evaluable cohorts | No |
| S2-079 | Malhotra et al. 2021 | NCT03026166 | Later-line combination | Rovalpituzumab tesirine + nivolumab +/- ipilimumab | DLL3 | Reported regimens | All-grade TEAE | 42/42 | Combined safety cohorts | No |
| S2-080 | Malhotra et al. 2021 | NCT03026166 | Later-line combination | Rovalpituzumab tesirine + nivolumab +/- ipilimumab | DLL3 | Reported regimens | Grade ≥3 TEAE | 38/42 | Combined safety cohorts | No |
| S2-081 | Malhotra et al. 2021 | NCT03026166 | Later-line combination | Rovalpituzumab tesirine + nivolumab +/- ipilimumab | DLL3 | Reported regimens | Serious TEAE | 32/42 | Combined safety cohorts | No |
| S2-082 | Malhotra et al. 2021 | NCT03026166 | Later-line combination | Rovalpituzumab tesirine + nivolumab +/- ipilimumab | DLL3 | Reported regimens | All-grade TRAE | 41/42 | Combined safety cohorts | No |
| S2-083 | Malhotra et al. 2021 | NCT03026166 | Later-line combination | Rovalpituzumab tesirine + nivolumab +/- ipilimumab | DLL3 | Reported regimens | Grade ≥3 TRAE | 27/42 | Combined safety cohorts | No |
| S2-084 | Malhotra et al. 2021 | NCT03026166 | Later-line combination | Rovalpituzumab tesirine + nivolumab +/- ipilimumab | DLL3 | Reported regimens | Serious TRAE | 19/42 | Combined safety cohorts | No |
| S2-085 | Udagawa et al. 2019 | NCT03086239 | Later-line monotherapy | Rovalpituzumab tesirine | DLL3 | 0.2 or 0.3 mg/kg | ORR | 3/29 | All treated SCLC patients | Yes |
| S2-086 | Udagawa et al. 2019 | NCT03086239 | Later-line monotherapy | Rovalpituzumab tesirine | DLL3 | 0.2 or 0.3 mg/kg | DCR | 16/29 | All treated SCLC patients | Yes |
| S2-087 | Udagawa et al. 2019 | NCT03086239 | Later-line monotherapy | Rovalpituzumab tesirine | DLL3 | 0.2 or 0.3 mg/kg | All-grade TEAE | 29/29 | Safety set | Yes |
| S2-088 | Udagawa et al. 2019 | NCT03086239 | Later-line monotherapy | Rovalpituzumab tesirine | DLL3 | 0.2 or 0.3 mg/kg | Serious TEAE | 13/29 | Safety set | Yes |
| S2-089 | Udagawa et al. 2019 | NCT03086239 | Later-line monotherapy | Rovalpituzumab tesirine | DLL3 | 0.2 or 0.3 mg/kg | All-grade TRAE | 29/29 | Safety set | Yes |
| S2-090 | Udagawa et al. 2019 | NCT03086239 | Later-line monotherapy | Rovalpituzumab tesirine | DLL3 | 0.2 or 0.3 mg/kg | Grade ≥3 TRAE | 15/29 | Safety set | Yes |
| S2-091 | Udagawa et al. 2019 | NCT03086239 | Later-line monotherapy | Rovalpituzumab tesirine | DLL3 | 0.2 or 0.3 mg/kg | Serious TRAE | 5/29 | Safety set | Yes |

**Table S2B.** Arm- and outcome-level data used in the quantitative synthesis: source and reconciliation fields.

| Record | Source/location | Reconciliation note |
| --- | --- | --- |
| S2-001 | Ma et al. 2025, main Table 3; online Supplementary Table 7, p. 21 | Direct numerator and denominator reported by source; Safety set was 79; efficacy denominator is 72.; Single reported arm or study-level aggregate |
| S2-002 | Ma et al. 2025, main Table 3; online Supplementary Table 7, p. 21 | Direct numerator and denominator reported by source; Single reported arm or study-level aggregate |
| S2-003 | Ma et al. 2025, online Supplementary Table 7, p. 21 | Sum of mutually exclusive dose or regimen cohorts; Two disjoint safety cohorts: 36 and 38 patients.; Recommended-dose safety subset; efficacy and safety use different analysis sets |
| S2-004 | Ma et al. 2025, online Supplementary Table 7, p. 21 | Sum of mutually exclusive dose or regimen cohorts; 18/36 and 27/38 combined; cohorts are disjoint.; Recommended-dose safety subset; efficacy and safety use different analysis sets |
| S2-005 | Ma et al. 2025, online Supplementary Table 7, p. 21 | Sum of mutually exclusive dose or regimen cohorts; 12/36 and 14/38 combined; cohorts are disjoint.; Recommended-dose safety subset; efficacy and safety use different analysis sets |
| S2-006 | Dowlati et al. 2025, main efficacy results | Direct numerator and denominator reported by source; Single reported arm or study-level aggregate |
| S2-007 | Dowlati et al. 2025, main efficacy results | Direct numerator and denominator reported by source; Single reported arm or study-level aggregate |
| S2-008 | Dowlati et al. 2025, safety results | Direct numerator and denominator reported by source; Single reported arm or study-level aggregate |
| S2-009 | Dowlati et al. 2025, safety results | Direct numerator and denominator reported by source; Single reported arm or study-level aggregate |
| S2-010 | Dowlati et al. 2025, safety results | Direct numerator and denominator reported by source; Single reported arm or study-level aggregate |
| S2-011 | Dowlati et al. 2025, safety results | Direct numerator and denominator reported by source; Single reported arm or study-level aggregate |
| S2-012 | Dowlati et al. 2025, safety results | Direct numerator and denominator reported by source; Single reported arm or study-level aggregate |
| S2-013 | Rudin et al. 2026, main efficacy results | Sum of mutually exclusive dose or regimen cohorts; 46 and 137 patient dose cohorts combined; no shared control.; Aggregate of mutually exclusive cohorts |
| S2-014 | Rudin et al. 2026, main efficacy results | Sum of mutually exclusive dose or regimen cohorts; Aggregate of mutually exclusive cohorts |
| S2-015 | Rudin et al. 2026, Data Supplement; Data Supplement Table S4 | Sum of mutually exclusive dose or regimen cohorts; 45/46 and 135/137 combined; cohorts are disjoint.; Aggregate of mutually exclusive cohorts |
| S2-016 | Rudin et al. 2026, Data Supplement; Data Supplement Table S4 | Sum of mutually exclusive dose or regimen cohorts; 21/46 and 85/137 combined; cohorts are disjoint.; Aggregate of mutually exclusive cohorts |
| S2-017 | Rudin et al. 2026, Data Supplement; main Table 5; Data Supplement Table S4 | Sum of mutually exclusive dose or regimen cohorts; 15/46 and 54/137 combined; cohorts are disjoint.; Aggregate of mutually exclusive cohorts |
| S2-018 | Rudin et al. 2026, Data Supplement; main Table 5; Data Supplement Table S4 | Sum of mutually exclusive dose or regimen cohorts; 35/46 and 123/137 combined; cohorts are disjoint.; Aggregate of mutually exclusive cohorts |
| S2-019 | Rudin et al. 2026, Data Supplement; main Table 5; Data Supplement Table S4 | Sum of mutually exclusive dose or regimen cohorts; 6/46 and 50/137 combined; cohorts are disjoint.; Aggregate of mutually exclusive cohorts |
| S2-020 | Rudin et al. 2026, Data Supplement; main Table 5; Data Supplement Table S4 | Sum of mutually exclusive dose or regimen cohorts; 3/46 and 25/137 combined; cohorts are disjoint.; Aggregate of mutually exclusive cohorts |
| S2-021 | Wang et al. 2024, J Clin Oncol 2024; 42(Suppl): abstract 8093 | Sum of mutually exclusive dose cohorts; confirmed responses only, with five responses awaiting confirmation excluded; Confirmed responses only; the reported 30/52 included five responses awaiting confirmation.; Aggregate of mutually exclusive cohorts |
| S2-022 | Wang et al. 2024, Poster efficacy table | Sum of mutually exclusive dose or regimen cohorts; 25/31 and 20/21 combined.; Aggregate of mutually exclusive cohorts |
| S2-023 | Morgensztern et al. 2024, efficacy table | Direct numerator and denominator reported by source; The nested 1-mg/kg cohort (n=40) is not added to this overall cohort.; Single reported arm or study-level aggregate |
| S2-024 | Morgensztern et al. 2024, efficacy table | Direct numerator and denominator reported by source; Single reported arm or study-level aggregate |
| S2-025 | Morgensztern et al. 2024, safety table | Direct numerator and denominator reported by source; Nested subset used only where the overall cohort outcome was not reported.; Nested recommended-dose subset; never added to the overall cohort for the same outcome |
| S2-026 | Morgensztern et al. 2024, safety table | Direct numerator and denominator reported by source; Nested recommended-dose subset; never added to the overall cohort for the same outcome |
| S2-027 | Morgensztern et al. 2024, safety table | Direct numerator and denominator reported by source; Nested recommended-dose subset; never added to the overall cohort for the same outcome |
| S2-028 | Morgensztern et al. 2024, safety table | Direct numerator and denominator reported by source; Nested recommended-dose subset; never added to the overall cohort for the same outcome |
| S2-029 | Morgensztern et al. 2024, safety table | Direct numerator and denominator reported by source; Nested recommended-dose subset; never added to the overall cohort for the same outcome |
| S2-030 | Morgensztern et al. 2024, safety table | Direct numerator and denominator reported by source; Nested recommended-dose subset; never added to the overall cohort for the same outcome |
| S2-031 | Blackhall et al. 2021, Table 2 | Direct numerator and denominator reported by source; Topotecan control excluded.; Single reported arm or study-level aggregate |
| S2-032 | Blackhall et al. 2021, Table 2 | Direct numerator and denominator reported by source; Topotecan control excluded.; Single reported arm or study-level aggregate |
| S2-033 | Blackhall et al. 2021, safety results | Direct numerator and denominator reported by source; Single reported arm or study-level aggregate |
| S2-034 | Blackhall et al. 2021, safety results | Direct numerator and denominator reported by source; Single reported arm or study-level aggregate |
| S2-035 | Blackhall et al. 2021, safety results | Direct numerator and denominator reported by source; Single reported arm or study-level aggregate |
| S2-036 | Morgensztern et al. 2020, efficacy results; p. 6 | Direct numerator and denominator reported by source; SCLC and LCNEC outcomes cannot be separated; excluded from the SCLC-only quantitative synthesis.; Single reported arm or study-level aggregate; SCLC and LCNEC outcomes could not be separated |
| S2-037 | Morgensztern et al. 2020, efficacy results; p. 6 | Direct numerator and denominator reported by source; SCLC and LCNEC outcomes cannot be separated.; Single reported arm or study-level aggregate; SCLC and LCNEC outcomes could not be separated |
| S2-038 | Morgensztern et al. 2020, Table 2; p. 12 | Direct numerator and denominator reported by source; SCLC and LCNEC outcomes cannot be separated.; Single reported arm or study-level aggregate; SCLC and LCNEC outcomes could not be separated |
| S2-039 | Morgensztern et al. 2020, Table 3; p. 13 | Direct numerator and denominator reported by source; SCLC and LCNEC outcomes cannot be separated.; Single reported arm or study-level aggregate; SCLC and LCNEC outcomes could not be separated |
| S2-040 | Morgensztern et al. 2020, Table 2; p. 12 | Direct numerator and denominator reported by source; SCLC and LCNEC outcomes cannot be separated.; Single reported arm or study-level aggregate; SCLC and LCNEC outcomes could not be separated |
| S2-041 | Morgensztern et al. 2020, Table 2; p. 12 | Direct numerator and denominator reported by source; SCLC and LCNEC outcomes cannot be separated.; Single reported arm or study-level aggregate; SCLC and LCNEC outcomes could not be separated |
| S2-042 | Morgensztern et al. 2020, Table 2; p. 12 | Direct numerator and denominator reported by source; SCLC and LCNEC outcomes cannot be separated.; Single reported arm or study-level aggregate; SCLC and LCNEC outcomes could not be separated |
| S2-043 | Morgensztern et al. 2019, efficacy results | Direct numerator and denominator reported by source; Single reported arm or study-level aggregate |
| S2-044 | Morgensztern et al. 2019, efficacy results | Direct numerator and denominator reported by source; Single reported arm or study-level aggregate |
| S2-045 | Morgensztern et al. 2019, safety table | Direct numerator and denominator reported by source; Single reported arm or study-level aggregate |
| S2-046 | Morgensztern et al. 2019, abstract and Results; pp. 2 and 7 | Direct numerator and denominator reported by source; The source's Table 3 maximum-grade row is internally inconsistent; 179 grade 3–4 plus 34 grade 5 equals 213.; Single reported arm or study-level aggregate |
| S2-047 | Morgensztern et al. 2019, results; p. 7 | Direct numerator and denominator reported by source; Corrected from the former mislabel 'serious TEAE'.; Single reported arm or study-level aggregate |
| S2-048 | Morgensztern et al. 2019, safety table | Direct numerator and denominator reported by source; Single reported arm or study-level aggregate |
| S2-049 | Morgensztern et al. 2019, Table 3; p. 19 | Direct numerator and denominator reported by source; Descriptive only; not pooled because 10 separately reported grade 5 drug-related events could not be combined into a non-overlapping grade ≥3 total.; Single reported arm or study-level aggregate; grade 3–4 TRAE; separately reported grade 5 drug-related events could not be combined into a non-overlapping grade ≥3 total |
| S2-050 | Calvo et al. 2021, Table 4; p. 5 | Direct numerator and denominator reported by source; The later full report supersedes the 2019 abstract; not pooled with monotherapy.; Single reported arm or study-level aggregate; Not part of the primary later-line monotherapy estimand: Later-line combination |
| S2-051 | Calvo et al. 2021, Table 4; p. 5 | Derived from mutually exclusive best-overall-response categories; Derived from 1 CR + 9 PR (best overall) + 12 SD; reported separately from confirmed ORR.; Single reported arm or study-level aggregate; Not part of the primary later-line monotherapy estimand: Later-line combination |
| S2-052 | Calvo et al. 2021, Table 3; p. 4 | Direct numerator and denominator reported by source; Single reported arm or study-level aggregate; Not part of the primary later-line monotherapy estimand: Later-line combination |
| S2-053 | Calvo et al. 2021, Table 3; p. 4 | Direct numerator and denominator reported by source; Single reported arm or study-level aggregate; Not part of the primary later-line monotherapy estimand: Later-line combination |
| S2-054 | Calvo et al. 2021, Table 3; p. 4 | Direct numerator and denominator reported by source; Single reported arm or study-level aggregate; Not part of the primary later-line monotherapy estimand: Later-line combination |
| S2-055 | Calvo et al. 2021, Table 3; p. 4 | Direct numerator and denominator reported by source; Single reported arm or study-level aggregate; Not part of the primary later-line monotherapy estimand: Later-line combination |
| S2-056 | Rudin et al. 2017, Table 3; p. 10 | Direct numerator and denominator reported by source; Single reported arm or study-level aggregate |
| S2-057 | Rudin et al. 2017, Table 3; p. 10 | Direct numerator and denominator reported by source; Single reported arm or study-level aggregate |
| S2-058 | Rudin et al. 2017, safety results; p. 9 | Direct numerator and denominator reported by source; Previously mislabeled as TEAE in the workbook.; Single reported arm or study-level aggregate |
| S2-059 | Rudin et al. 2017, safety results; p. 9 | Direct numerator and denominator reported by source; Previously mislabeled as TEAE in the workbook.; Single reported arm or study-level aggregate |
| S2-060 | Rudin et al. 2017, abstract; p. 2 | Direct numerator and denominator reported by source; The main report and its mixed-histology supplement give internally conflicting serious-related counts; retained descriptively but excluded from pooling.; Single reported arm or study-level aggregate; Conflicting serious TRAE counts across the abstract, main text, and supplement |
| S2-061 | Gray et al. 2017, Table 3; p. 6 | Direct numerator and denominator reported by source; Single reported arm or study-level aggregate |
| S2-062 | Gray et al. 2017, best-response results and Table 3; pp. 4–6 | Derived from mutually exclusive best-overall-response categories; Derived directly as 7 PR + 21 SD. The duration-restricted CBR was 17/50 and was not used as DCR.; Single reported arm or study-level aggregate |
| S2-063 | Johnson et al. 2021, efficacy results; p. 6 | Direct numerator and denominator reported by source; Placebo excluded; not pooled with frontline or later-line treatment.; Single reported arm or study-level aggregate; Not part of the primary later-line monotherapy estimand: Post-induction maintenance |
| S2-064 | Johnson et al. 2021, efficacy results; p. 6 | Direct numerator and denominator reported by source; Single reported arm or study-level aggregate; Not part of the primary later-line monotherapy estimand: Post-induction maintenance |
| S2-065 | Johnson et al. 2021, safety results; p. 6 | Direct numerator and denominator reported by source; Single reported arm or study-level aggregate; Not part of the primary later-line monotherapy estimand: Post-induction maintenance |
| S2-066 | Johnson et al. 2021, safety results; p. 7 | Direct numerator and denominator reported by source; Single reported arm or study-level aggregate; Not part of the primary later-line monotherapy estimand: Post-induction maintenance |
| S2-067 | Johnson et al. 2021, safety results; p. 7 | Direct numerator and denominator reported by source; Single reported arm or study-level aggregate; Not part of the primary later-line monotherapy estimand: Post-induction maintenance |
| S2-068 | Johnson et al. 2021, safety results; p. 7 | Direct numerator and denominator reported by source; Single reported arm or study-level aggregate; Not part of the primary later-line monotherapy estimand: Post-induction maintenance |
| S2-069 | Socinski et al. 2017, efficacy results | Reconstructed from rounded source percentage and reported denominator; Reconstructed from the source-reported rounded percentage and response-evaluable denominator; control chemotherapy arm excluded.; Single reported arm or study-level aggregate; Not part of the primary later-line monotherapy estimand: Frontline combination |
| S2-070 | Socinski et al. 2017, efficacy results | Reconstructed from rounded source percentage and reported denominator; Reconstructed from the source-reported rounded percentage and response-evaluable denominator.; Single reported arm or study-level aggregate; Not part of the primary later-line monotherapy estimand: Frontline combination |
| S2-071 | Socinski et al. 2017, safety table | Direct numerator and denominator reported by source; Single reported arm or study-level aggregate; Not part of the primary later-line monotherapy estimand: Frontline combination |
| S2-072 | Socinski et al. 2017, safety table | Direct numerator and denominator reported by source; Single reported arm or study-level aggregate; Not part of the primary later-line monotherapy estimand: Frontline combination |
| S2-073 | Socinski et al. 2017, safety results | Direct numerator and denominator reported by source; Single reported arm or study-level aggregate; Not part of the primary later-line monotherapy estimand: Frontline combination |
| S2-074 | Hann et al. 2021, Table 3; p. 5 | Direct numerator and denominator reported by source; Cohorts 1, 2, and 4 were mixed monotherapy/sequential/maintenance contexts and were not pooled.; Single reported arm or study-level aggregate; Not part of the primary later-line monotherapy estimand: Frontline combination |
| S2-075 | Hann et al. 2021, safety results; p. 3 | Direct numerator and denominator reported by source; Single reported arm or study-level aggregate; Not part of the primary later-line monotherapy estimand: Frontline combination |
| S2-076 | Hann et al. 2021, safety results; p. 3 | Direct numerator and denominator reported by source; Single reported arm or study-level aggregate; Not part of the primary later-line monotherapy estimand: Frontline combination |
| S2-077 | Hann et al. 2021, safety results; p. 4 | Direct numerator and denominator reported by source; Single reported arm or study-level aggregate; Not part of the primary later-line monotherapy estimand: Frontline combination |
| S2-078 | Malhotra et al. 2021, Table 3; p. 8 | Sum of mutually exclusive dose or regimen cohorts; 8/29 and 4/11; the treated denominator 42 is not used for ORR.; Aggregate of mutually exclusive cohorts; Not part of the primary later-line monotherapy estimand: Later-line combination |
| S2-079 | Malhotra et al. 2021, safety results; p. 5 | Sum of mutually exclusive dose or regimen cohorts; Aggregate of mutually exclusive cohorts; Not part of the primary later-line monotherapy estimand: Later-line combination |
| S2-080 | Malhotra et al. 2021, safety results; p. 5 | Sum of mutually exclusive dose or regimen cohorts; Aggregate of mutually exclusive cohorts; Not part of the primary later-line monotherapy estimand: Later-line combination |
| S2-081 | Malhotra et al. 2021, Table 2; p. 6 | Sum of mutually exclusive dose or regimen cohorts; Aggregate of mutually exclusive cohorts; Not part of the primary later-line monotherapy estimand: Later-line combination |
| S2-082 | Malhotra et al. 2021, safety results; p. 5 | Sum of mutually exclusive dose or regimen cohorts; Aggregate of mutually exclusive cohorts; Not part of the primary later-line monotherapy estimand: Later-line combination |
| S2-083 | Malhotra et al. 2021, safety results; p. 5 | Sum of mutually exclusive dose or regimen cohorts; Aggregate of mutually exclusive cohorts; Not part of the primary later-line monotherapy estimand: Later-line combination |
| S2-084 | Malhotra et al. 2021, safety results; p. 5 | Sum of mutually exclusive dose or regimen cohorts; Aggregate of mutually exclusive cohorts; Not part of the primary later-line monotherapy estimand: Later-line combination |
| S2-085 | Udagawa et al. 2019, Table 4; p. 22 | Direct numerator and denominator reported by source; Single reported arm or study-level aggregate |
| S2-086 | Udagawa et al. 2019, Table 4; p. 22 | Direct numerator and denominator reported by source; Single reported arm or study-level aggregate |
| S2-087 | Udagawa et al. 2019, safety results; p. 10 | Direct numerator and denominator reported by source; Single reported arm or study-level aggregate |
| S2-088 | Udagawa et al. 2019, safety results; p. 10 | Direct numerator and denominator reported by source; Single reported arm or study-level aggregate |
| S2-089 | Udagawa et al. 2019, Table 2; p. 21 | Direct numerator and denominator reported by source; Grade categories sum to 29.; Single reported arm or study-level aggregate |
| S2-090 | Udagawa et al. 2019, Table 2; p. 21 | Direct numerator and denominator reported by source; 11 grade 3 and 4 grade 4.; Single reported arm or study-level aggregate |
| S2-091 | Udagawa et al. 2019, safety results; p. 10 | Direct numerator and denominator reported by source; Single reported arm or study-level aggregate |

Tables S2A and S2B use the same record identifier. Table S2A reports the molecular target, endpoint-specific cohort, numerator, denominator, analysis population, and inclusion decision; Table S2B reports the external source citation, reproducible table/section/page location, and reconciliation details. DCR values derived directly from reported best-response categories are identified in the notes and are not described as verbatim author-reported DCR counts.

**Table S3.** Records excluded from quantitative synthesis and reasons.

| **Item** | **Decision** | **Reason** |
| --- | --- | --- |
| TAHOE topotecan arm | Excluded | Control arm; quantitative estimand is an ADC-arm proportion. |
| MERU placebo arm | Excluded | Control arm; quantitative estimand is an ADC-arm proportion. |
| Socinski carboplatin/etoposide control arm | Excluded | Control arm. |
| SC-002 mixed SCLC/LCNEC cohort | Excluded from SCLC quantitative synthesis | The article pooled 35 patients with SCLC or LCNEC and did not report histology-specific denominators or outcomes. |
| Calvo 2019 conference abstract | Superseded | Replaced by the later 2021 peer-reviewed full report of the same trial family; the two reports are not counted twice. |
| ABBV-011 1-mg/kg efficacy subset (n=40) | Excluded from efficacy pooling | Nested within the overall ABBV-011 cohort; adding both would double count patients. |
| Hann cohorts 1, 2, and 4 | Excluded from pooled frontline estimate | Mixed monotherapy, sequential, and maintenance contexts are not exchangeable with concurrent chemotherapy cohort 3. |
| Fixed-time 6/12-month PFS and OS | Removed from quantitative synthesis | Available values were KM probabilities or risk-set values without consistently reported censoring-aware SEs/CIs; they cannot be pooled as ordinary x/n data. |
| Common adverse-event incidence meta-analyses | Removed | Selective reporting thresholds, outcome-label errors, and heterogeneous regimen contexts made pooled incidence potentially misleading; exact reported counts remain in source tables. |
| Funnel plots and Egger/Begg tests | Removed | Conventional asymmetry methods are not validated for sparse single-arm proportions and most analyses had too few studies. |

**Table S4.** Methodological index for non-randomized studies (MINORS).

| **Study** | **Q1** | **Q2** | **Q3** | **Q4** | **Q5** | **Q6** | **Q7** | **Q8** | **Score** | **Category** |
| --- | --- | --- | --- | --- | --- | --- | --- | --- | --- | --- |
| Ma et al. 2025 | 2 | 2 | 2 | 2 | 1 | 1 | 0 | 2 | 12 | Intermediate |
| Dowlati et al. 2025 | 2 | 2 | 2 | 2 | 1 | 2 | 0 | 0 | 11 | Intermediate |
| Rudin et al. 2026 | 2 | 2 | 2 | 2 | 1 | 2 | 0 | 2 | 13 | High |
| Morgensztern et al. 2024 | 2 | 2 | 2 | 2 | 1 | 2 | 2 | 0 | 13 | High |
| Wang et al. 2024 | 2 | 2 | 2 | 2 | 1 | 2 | 1 | 2 | 14 | High |
| Morgensztern et al. 2020 | 2 | 2 | 2 | 2 | 1 | 2 | 2 | 1 | 14 | High |
| Morgensztern et al. 2019 | 2 | 2 | 2 | 2 | 1 | 2 | 1 | 2 | 14 | High |
| Calvo et al. 2021 | 2 | 2 | 2 | 2 | 1 | 2 | 1 | 1 | 13 | High |
| Rudin et al. 2017 | 2 | 2 | 2 | 2 | 1 | 2 | 1 | 1 | 13 | High |
| Gray et al. 2017 | 2 | 2 | 2 | 2 | 2 | 2 | 0 | 0 | 12 | Intermediate |
| Malhotra et al. 2021 | 2 | 2 | 2 | 2 | 2 | 2 | 0 | 0 | 12 | Intermediate |
| Udagawa et al. 2019 | 2 | 2 | 2 | 2 | 1 | 2 | 0 | 0 | 11 | Intermediate |
| Hann et al. 2021 | 2 | 2 | 2 | 2 | 1 | 2 | 0 | 0 | 11 | Intermediate |

Items are scored 0 (not reported), 1 (reported but inadequate), or 2 (reported and adequate). For non-comparative studies, the prespecified descriptive categories were >12, high; 8–12, intermediate; and <8, low. Seven studies were in the high category and six in the intermediate category. These categories are not outcome-level certainty ratings.

**Table S5.** Result-specific RoB 2 scope, domain judgments, and published-report rationale.

| **Study** | **Comparison** | **Outcome** | **Time point** | **Estimand** |
| --- | --- | --- | --- | --- |
| Blackhall et al. 2021 | Rovalpituzumab tesirine vs topotecan | Overall survival | Final analysis; data cutoff 12 February 2020 | Effect of assignment to intervention |
| Johnson et al. 2021 | Rovalpituzumab tesirine vs placebo | Overall survival in DLL3-high randomized patients | Protocol-defined futility analysis | Effect of assignment to intervention |
| Socinski et al. 2017 | Lorvotuzumab mertansine plus carboplatin/etoposide vs carboplatin/etoposide | Progression-free survival | Final phase 2 analysis | Effect of assignment to intervention |

Domain-level judgments and rationales follow.

| **Study** | **Domain** | **Judgment** | **Published-report signalling basis** |
| --- | --- | --- | --- |
| Blackhall et al. 2021 | Randomization process | Some concerns | Stratified 2:1 randomization was reported, but sequence generation and allocation concealment were not described in the publication. |
| Blackhall et al. 2021 | Deviations from intended interventions | Some concerns | The trial was open label and dose modification or treatment beyond progression was permitted; OS was analyzed in all randomized patients. |
| Blackhall et al. 2021 | Missing outcome data | Low risk | OS used all randomized patients with prespecified censoring; only limited loss to follow-up was evident in the report. |
| Blackhall et al. 2021 | Measurement of the outcome | Low risk | Death is an objective outcome and was measured from randomization. |
| Blackhall et al. 2021 | Selection of the reported result | Some concerns | OS was reported as the prespecified primary endpoint, but the protocol and statistical analysis plan were unavailable to verify the choice among eligible analyses and time points. |
| Blackhall et al. 2021 | Overall | Some concerns | Some concerns remain because allocation procedures and prespecified result-selection choices could not be fully verified from the available report, and the trial was open label. |
| Johnson et al. 2021 | Randomization process | Some concerns | Stratified 1:1 randomization was reported, but sequence generation and allocation concealment were not described in the publication. |
| Johnson et al. 2021 | Deviations from intended interventions | Low risk | Double blinding and matched placebo were reported; OS was analyzed in the randomized DLL3-high population. |
| Johnson et al. 2021 | Missing outcome data | Low risk | OS was available for the randomized analysis with very limited loss to follow-up reported. |
| Johnson et al. 2021 | Measurement of the outcome | Low risk | Death is an objective outcome and was measured from randomization. |
| Johnson et al. 2021 | Selection of the reported result | Some concerns | OS in DLL3-high patients and the futility analysis were described as prespecified, but the protocol and statistical analysis plan were unavailable to verify the choice among eligible analyses, time points, and subgroups. |
| Johnson et al. 2021 | Overall | Some concerns | Some concerns remain because allocation procedures and prespecified result-selection choices could not be fully verified from the available report. |
| Socinski et al. 2017 | Randomization process | Some concerns | A 2:1 randomized allocation was reported, but sequence generation, concealment, and stratification were not described. |
| Socinski et al. 2017 | Deviations from intended interventions | Some concerns | The trial was open label, dosing was amended, and post-combination single-agent treatment was permitted in the experimental arm. |
| Socinski et al. 2017 | Missing outcome data | Some concerns | The efficacy population required a postbaseline assessment rather than including every randomized patient. |
| Socinski et al. 2017 | Measurement of the outcome | Some concerns | Progression was investigator assessed in an open-label trial without reported blinded central review. |
| Socinski et al. 2017 | Selection of the reported result | Some concerns | The study was not powered for a randomized PFS comparison and the publication did not provide a protocol or analysis plan for verification. |
| Socinski et al. 2017 | Overall | High risk | Multiple domains raised concerns, including exclusion from the efficacy population, open-label outcome assessment, protocol amendments, and incomplete reporting of the comparative analysis plan. |

RoB 2 judgments apply only to the explicitly stated randomized comparative result and to the effect of assignment to intervention. They do not assess bias in the single-arm ADC-arm proportions used in the primary synthesis and do not constitute certainty-of-evidence ratings. Protocols and statistical analysis plans were not available; judgments were therefore based on the published reports.

**Table S6.** Primary and sensitivity meta-analytic models for later-line ADC monotherapy.

| **Outcome** | **Model** | **k** | **Events/N** | **Estimate (95% CI)** | **τ² (logit)** | **I²** | **95% prediction interval** |
| --- | --- | --- | --- | --- | --- | --- | --- |
| All-grade TEAE | Primary: binomial-normal random-intercept GLMM (ML) | 6 | 899/921 | 98.1% (95.9%–99.2%) | 0.322 | 38.3% | 89.7%–99.7% |
| All-grade TRAE | Primary: binomial-normal random-intercept GLMM (ML) | 7 | 706/782 | 93.0% (85.0%–96.9%) | 0.822 | 60.0% | 53.3%–99.4% |
| DCR | Primary: binomial-normal random-intercept GLMM (ML) | 10 | 796/1213 | 72.7% (60.7%–82.2%) | 0.691 | 94.5% | 26.8%–95.1% |
| Grade ≥3 TEAE | Primary: binomial-normal random-intercept GLMM (ML) | 5 | 560/892 | 62.8% (59.6%–65.9%) | 0.000 | 10.6% | 58.2%–67.2% |
| Grade ≥3 TRAE | Primary: binomial-normal random-intercept GLMM (ML) | 6 | 185/443 | 45.5% (35.5%–56.0%) | 0.198 | 81.8% | 19.0%–74.8% |
| ORR | Primary: binomial-normal random-intercept GLMM (ML) | 10 | 291/1213 | 25.7% (16.4%–37.9%) | 0.746 | 93.8% | 4.2%–73.1% |
| Serious TEAE | Primary: binomial-normal random-intercept GLMM (ML) | 5 | 282/582 | 47.1% (39.7%–54.7%) | 0.063 | 73.1% | 28.2%–66.9% |
| Serious TRAE | Primary: binomial-normal random-intercept GLMM (ML) | 5 | 162/665 | 20.5% (12.9%–31.0%) | 0.285 | 82.5% | 4.6%–57.9% |
| All-grade TEAE | Sensitivity: inverse-variance logit, REML/Hartung-Knapp | 6 | 899/921 | 97.7% (95.5%–98.9%) | 0.325 | 43.2% | 88.3%–99.6% |
| All-grade TRAE | Sensitivity: inverse-variance logit, REML/Hartung-Knapp | 7 | 706/782 | 90.6% (79.4%–96.0%) | 0.421 | 64.7% | 62.1%–98.3% |
| DCR | Sensitivity: inverse-variance logit, REML/Hartung-Knapp | 10 | 796/1213 | 72.3% (57.6%–83.4%) | 0.744 | 94.5% | 25.0%–95.3% |
| Grade ≥3 TEAE | Sensitivity: inverse-variance logit, REML/Hartung-Knapp | 5 | 560/892 | 62.7% (57.8%–67.3%) | 0.000 | 10.6% | 58.1%–67.1% |
| Grade ≥3 TRAE | Sensitivity: inverse-variance logit, REML/Hartung-Knapp | 6 | 185/443 | 45.7% (32.1%–59.9%) | 0.241 | 81.8% | 17.3%–77.2% |
| ORR | Sensitivity: inverse-variance logit, REML/Hartung-Knapp | 10 | 291/1213 | 26.1% (15.2%–41.0%) | 0.808 | 93.8% | 4.0%–75.1% |
| Serious TEAE | Sensitivity: inverse-variance logit, REML/Hartung-Knapp | 5 | 282/582 | 47.1% (37.5%–56.9%) | 0.082 | 73.1% | 26.2%–69.1% |
| Serious TRAE | Sensitivity: inverse-variance logit, REML/Hartung-Knapp | 5 | 162/665 | 20.9% (9.9%–38.7%) | 0.350 | 82.5% | 4.0%–62.6% |
| DCR | Sensitivity: recommended/expansion efficacy cohorts, GLMM (ML) | 10 | 713/1104 | 72.9% (60.2%–82.7%) | 0.760 | 94.2% | 25.1%–95.6% |
| ORR | Sensitivity: recommended/expansion efficacy cohorts, GLMM (ML) | 10 | 268/1104 | 26.9% (17.0%–39.9%) | 0.801 | 94.0% | 4.2%–75.7% |

Primary models were binomial–normal random-intercept generalized linear mixed models on the logit scale, fitted by maximum likelihood without continuity correction; pooled 95% CIs were classical Wald z intervals. Prediction intervals used the Veroniki method V with a t distribution and k−1 degrees of freedom. The inverse-variance sensitivity models used logit-transformed proportions, REML τ², and Hartung–Knapp pooled CIs; 0.5 was added only for zero-event or all-event boundary proportions, and prediction intervals again used method V. τ² is reported on the logit scale. The recommended/expansion sensitivity replaces selected all-dose cohorts with clinically relevant recommended or expansion cohorts; nested representations are never included simultaneously.

**Table S7.** Context-specific descriptive estimates outside the primary later-line monotherapy meta-analysis.

| **Study/report** | **Clinical context** | **Regimen** | **Outcome** | **Events/N** | **Exact estimate (95% CI)** | **Analysis set** | **Interpretive status** |
| --- | --- | --- | --- | --- | --- | --- | --- |
| Socinski et al. 2017 | Frontline combination | Lorvotuzumab mertansine + carboplatin/etoposide | DCR | 75/82 | 91.5% (83.2%–96.5%) | Response-evaluable experimental arm | Not part of the primary later-line monotherapy estimand: Frontline combination |
| Calvo et al. 2021 | Later-line combination | Rovalpituzumab tesirine + budigalimab | DCR | 22/29 | 75.9% (56.5%–89.7%) | Efficacy-evaluable set | Not part of the primary later-line monotherapy estimand: Later-line combination |
| Morgensztern et al. 2020 | Later-line mixed histology | SC-002 | DCR | 19/35 | 54.3% (36.6%–71.2%) | Combined SCLC/LCNEC efficacy set | SCLC and LCNEC outcomes could not be separated |
| Johnson et al. 2021 | Post-induction maintenance | Rovalpituzumab tesirine | DCR | 211/318 | 66.4% (60.9%–71.5%) | Response-evaluable Rova-T arm | Not part of the primary later-line monotherapy estimand: Post-induction maintenance |
| Morgensztern et al. 2019 | Later-line monotherapy | Rovalpituzumab tesirine | grade 3–4 TRAE | 135/339 | 39.8% (34.6%–45.3%) | Safety set | grade 3–4 TRAE; separately reported grade 5 drug-related events could not be combined into a non-overlapping grade ≥3 total |
| Hann et al. 2021 | Frontline combination | Rovalpituzumab tesirine + concurrent platinum/etoposide | Grade ≥3 TEAE | 13/14 | 92.9% (66.1%–99.8%) | Cohort 3 | Not part of the primary later-line monotherapy estimand: Frontline combination |
| Socinski et al. 2017 | Frontline combination | Lorvotuzumab mertansine + carboplatin/etoposide | Grade ≥3 TEAE | 92/94 | 97.9% (92.5%–99.7%) | Experimental-arm safety set | Not part of the primary later-line monotherapy estimand: Frontline combination |
| Calvo et al. 2021 | Later-line combination | Rovalpituzumab tesirine + budigalimab | Grade ≥3 TEAE | 26/31 | 83.9% (66.3%–94.5%) | Safety set | Not part of the primary later-line monotherapy estimand: Later-line combination |
| Malhotra et al. 2021 | Later-line combination | Rovalpituzumab tesirine + nivolumab +/- ipilimumab | Grade ≥3 TEAE | 38/42 | 90.5% (77.4%–97.3%) | Combined safety cohorts | Not part of the primary later-line monotherapy estimand: Later-line combination |
| Morgensztern et al. 2020 | Later-line mixed histology | SC-002 | Grade ≥3 TEAE | 24/35 | 68.6% (50.7%–83.1%) | Combined SCLC/LCNEC safety set | SCLC and LCNEC outcomes could not be separated |
| Johnson et al. 2021 | Post-induction maintenance | Rovalpituzumab tesirine | Grade ≥3 TEAE | 217/368 | 59.0% (53.8%–64.0%) | Rova-T safety set | Not part of the primary later-line monotherapy estimand: Post-induction maintenance |
| Calvo et al. 2021 | Later-line combination | Rovalpituzumab tesirine + budigalimab | Grade ≥3 TRAE | 17/31 | 54.8% (36.0%–72.7%) | Safety set | Not part of the primary later-line monotherapy estimand: Later-line combination |
| Malhotra et al. 2021 | Later-line combination | Rovalpituzumab tesirine + nivolumab +/- ipilimumab | Grade ≥3 TRAE | 27/42 | 64.3% (48.0%–78.4%) | Combined safety cohorts | Not part of the primary later-line monotherapy estimand: Later-line combination |
| Hann et al. 2021 | Frontline combination | Rovalpituzumab tesirine + concurrent platinum/etoposide | ORR | 7/14 | 50.0% (23.0%–77.0%) | Cohort 3 | Not part of the primary later-line monotherapy estimand: Frontline combination |
| Socinski et al. 2017 | Frontline combination | Lorvotuzumab mertansine + carboplatin/etoposide | ORR | 55/82 | 67.1% (55.8%–77.1%) | Response-evaluable experimental arm | Not part of the primary later-line monotherapy estimand: Frontline combination |
| Calvo et al. 2021 | Later-line combination | Rovalpituzumab tesirine + budigalimab | ORR | 7/29 | 24.1% (10.3%–43.5%) | Efficacy-evaluable set | Not part of the primary later-line monotherapy estimand: Later-line combination |
| Malhotra et al. 2021 | Later-line combination | Rovalpituzumab tesirine + nivolumab +/- ipilimumab | ORR | 12/40 | 30.0% (16.6%–46.5%) | Combined efficacy-evaluable cohorts | Not part of the primary later-line monotherapy estimand: Later-line combination |
| Morgensztern et al. 2020 | Later-line mixed histology | SC-002 | ORR | 5/35 | 14.3% (4.8%–30.3%) | Combined SCLC/LCNEC efficacy set | SCLC and LCNEC outcomes could not be separated |
| Johnson et al. 2021 | Post-induction maintenance | Rovalpituzumab tesirine | ORR | 28/318 | 8.8% (5.9%–12.5%) | Response-evaluable Rova-T arm | Not part of the primary later-line monotherapy estimand: Post-induction maintenance |

These estimates are descriptive and are not pooled with the primary later-line monotherapy estimand. The TRINITY value of 135/339 is grade 3–4 TRAE, not grade ≥3 TRAE; it was not pooled because 10 grade 5 drug-related events were reported separately and a non-overlapping grade ≥3 total could not be derived. SC-002 combines SCLC and LCNEC and is retained only as a mixed-histology evidence record.

**Table S8.** Exploratory target-stratified summaries restricted to later-line ADC monotherapy.

| **Outcome** | **Target** | **k** | **Events/N** | **Estimate (95% CI)** | **τ²** | **I²** | **95% prediction interval** | **Synthesis** |
| --- | --- | --- | --- | --- | --- | --- | --- | --- |
| DCR | B7-H3 | 3 | 268/307 | 87.3% (83.1%–90.6%) | 0.000 | 0.0% | 76.7%–93.5% | Exploratory GLMM; no between-target comparison |
| DCR | DLL3 | 4 | 396/715 | 57.4% (42.3%–71.2%) | 0.329 | 95.9% | 14.5%–91.5% | Exploratory GLMM; no between-target comparison |
| DCR | SEZ6 | 1 | 68/98 | Not pooled | NR | NR | NR | Not pooled (k < 3) |
| DCR | Trop-2 | 2 | 64/93 | Not pooled | NR | NR | NR | Not pooled (k < 3) |
| ORR | B7-H3 | 3 | 149/307 | 50.9% (40.2%–61.5%) | 0.095 | 78.1% | 16.9%–84.1% | Exploratory GLMM; no between-target comparison |
| ORR | DLL3 | 4 | 98/715 | 13.7% (11.4%–16.4%) | 0.000 | 0.0% | 10.1%–18.3% | Exploratory GLMM; no between-target comparison |
| ORR | SEZ6 | 1 | 19/98 | Not pooled | NR | NR | NR | Not pooled (k < 3) |
| ORR | Trop-2 | 2 | 25/93 | Not pooled | NR | NR | NR | Not pooled (k < 3) |

No between-target test or ranking was performed. Targets represented by fewer than three independent studies were not pooled. Low or zero I² estimates based on few studies should not be interpreted as proof of homogeneity.

**Table S9.** Structured bias considerations for cohorts contributing to the primary single-arm synthesis.

| **Study/report** | **Pooled outcomes** | **Selection/enrolment** | **Analysis population/missing outcomes** | **Outcome ascertainment** | **Definition/timing** | **Selective reporting** | **Dose/cohort representativeness** |
| --- | --- | --- | --- | --- | --- | --- | --- |
| Ma et al. 2025 | All-grade TRAE; DCR; Grade ≥3 TRAE; ORR; Serious TRAE | Some concern: early-phase eligibility and organ-function requirements limit generalizability. | Some concern: efficacy used 72 evaluable patients rather than all 79 treated patients; safety outcomes used endpoint-specific recommended-dose populations. | Some concern: tumor response was assessed locally by investigators using RECIST 1.1. | Lower concern: response definitions and trial endpoints were specified in the full report. | Some concern: the report was from an early-phase program and not every safety outcome was available for every cohort. | Some concern: efficacy combined studied doses, whereas several safety outcomes used recommended-dose cohorts; recommended-dose efficacy was examined separately in sensitivity analysis. |
| Dowlati et al. 2025 | All-grade TEAE; All-grade TRAE; DCR; Grade ≥3 TEAE; Grade ≥3 TRAE; ORR; Serious TEAE | Some concern: a phase 2 second-line trial population may not represent all later-line patients. | Lower concern: efficacy and safety were analyzed in the 43-patient intention-to-treat population. | Some concern: the pooled ORR was investigator assessed, although blinded independent central review produced a similar estimate. | Lower concern: RECIST 1.1, confirmation, and scheduled imaging were reported. | Some concern: protocol and statistical analysis plan information was insufficient for a complete assessment of selective reporting. | Lower concern: all patients received the same 10-mg/kg regimen. |
| Rudin et al. 2026 | All-grade TEAE; All-grade TRAE; DCR; Grade ≥3 TEAE; Grade ≥3 TRAE; ORR; Serious TEAE; Serious TRAE | Some concern: trial eligibility and prior-treatment requirements restrict generalizability. | Lower concern: all 183 treated patients were included in the combined-dose efficacy and safety summaries. | Lower concern: the primary response outcome was assessed by blinded independent central review using RECIST 1.1. | Lower concern: imaging was scheduled every 6 weeks for 36 weeks and every 12 weeks thereafter. | Some concern: complete prespecification could not be verified without the full protocol and statistical analysis plan. | Some concern: the primary representation combined disjoint 8- and 12-mg/kg cohorts; a 12-mg/kg analysis was examined in sensitivity analysis. |
| Wang et al. 2024 | DCR; ORR | Some concern: early-phase dose-expansion eligibility limits generalizability. | High concern: 52 of 56 treated patients were efficacy evaluable, and the conference abstract provided limited information about exclusions. | High concern: the abstract did not report blinded independent response assessment. | Some concern: RECIST-based response was reported, but assessment timing and handling details were limited in the abstract. | High concern: only a conference abstract was available, with incomplete outcome and methods reporting. | Some concern: disjoint 8- and 10-mg/kg expansion cohorts were combined. |
| Morgensztern et al. 2024 | All-grade TEAE; All-grade TRAE; DCR; Grade ≥3 TEAE; Grade ≥3 TRAE; ORR; Serious TEAE; Serious TRAE | Some concern: dose-escalation and biomarker-selected expansion cohorts limit generalizability. | Some concern: 98 of 99 treated patients contributed to the response denominator; one patient without a postbaseline assessment was excluded. | Some concern: response was investigator assessed in an open-label study. | Lower concern: RECIST 1.1 and scheduled imaging every 6 weeks, then every 12 weeks, were reported. | Some concern: complete prespecification could not be verified from the publication alone. | High concern: efficacy combined several dose levels, whereas some safety outcomes came from the nested 1-mg/kg cohort; the 1-mg/kg efficacy cohort was examined separately in sensitivity analysis. |
| Blackhall et al. 2021 | All-grade TEAE; DCR; Grade ≥3 TEAE; ORR; Serious TEAE | Some concern: trial eligibility limits generalizability of the absolute ADC-arm proportion despite randomized allocation. | Lower concern: all 287 patients in the ADC arm were included, and patients without postbaseline imaging were treated as nonresponders. | Some concern: response was investigator assessed in an open-label trial. | Lower concern: RECIST 1.1 and a prespecified imaging schedule were reported. | Some concern: protocols and statistical analysis plans were unavailable for complete verification of result selection. | Lower concern: the ADC arm used a fixed 0.3-mg/kg regimen. |
| Morgensztern et al. 2019 | All-grade TEAE; All-grade TRAE; DCR; Grade ≥3 TEAE; ORR; Serious TRAE | Some concern: the study enrolled a biomarker-selected, third-line-or-later population. | Lower concern: all 339 treated patients contributed to the main efficacy and safety denominators. | Lower concern: the primary response outcome was assessed by central radiographic review using RECIST 1.1. | Lower concern: co-primary and secondary endpoints and scheduled assessments were reported. | Some concern: grade 3–4 treatment-related events and grade 5 drug-related events were reported separately; a non-overlapping grade ≥3 TRAE total could not be derived. | Lower concern: the reported cohort received a fixed 0.3-mg/kg regimen. |
| Rudin et al. 2017 | All-grade TRAE; DCR; Grade ≥3 TRAE; ORR | Some concern: a phase 1 dose-escalation/expansion population limits generalizability. | High concern: efficacy used 60 assessable active-dose patients rather than all 74 treated SCLC patients; early discontinuation before postbaseline imaging led to non-evaluable status. | Some concern: dedicated site radiology was used, with independent review only when available. | Lower concern: confirmed RECIST 1.1 responses and confirmation timing were reported. | Some concern: complete prespecification and the availability of all outcome definitions could not be verified from the report alone. | High concern: several active dose levels and schedules were combined. |
| Gray et al. 2017 | DCR; ORR | Some concern: a small phase 1/2 population with prior-treatment requirements limits generalizability. | Lower concern: ORR and DCR used the 50-patient intention-to-treat population, including seven patients without postbaseline imaging as nonresponders. | Some concern: response was assessed by site or contracted local radiology rather than a blinded central review. | Lower concern: RECIST 1.1, approximately 8-week imaging, and response confirmation were reported. | Some concern: complete protocol-based verification of result selection was unavailable. | Some concern: the 8- and 10-mg/kg cohorts were combined. |
| Udagawa et al. 2019 | All-grade TEAE; All-grade TRAE; DCR; Grade ≥3 TRAE; ORR; Serious TEAE; Serious TRAE | Some concern: a small Japanese phase 1 dose-escalation/expansion cohort limits generalizability. | Lower concern: all 29 treated patients contributed to the efficacy and safety denominators. | Some concern: both investigator and central-reader assessments were reported, but the report did not fully resolve which assessment supported every extracted response count. | Lower concern: RECIST 1.1 and scheduled imaging every 6 weeks, then every 12 weeks, were reported. | Some concern: complete prespecification could not be verified from the report alone. | Some concern: 0.2- and 0.3-mg/kg dose cohorts were combined. |

These considerations apply to the absolute ADC-arm proportions used in the primary synthesis. They are based on the published reports, are not an overall risk-of-bias score, and are not certainty ratings. The concern descriptors apply to individual domains; no overall rating was assigned.

# Supplementary figures

**Figure S1.** Report-based RoB 2 judgments for three explicitly scoped randomized comparative results: final overall survival in TAHOE, DLL3-high overall survival at the protocol-defined MERU futility analysis, and final phase 2 progression-free survival in IMGN901. D1, randomization process; D2, deviations from intended interventions; D3, missing outcome data; D4, measurement of the outcome; D5, selection of the reported result. The estimand was the effect of assignment to intervention. Domain rationales are in Table S5. These judgments do not assess bias in the single-arm ADC proportions used in the primary meta-analysis.

**Figure S2.** Leave-one-study-out analysis for objective response rate in later-line ADC monotherapy. The burgundy vertical line denotes the full-data primary GLMM estimate.

**Figure S3.** Leave-one-study-out analysis for disease control rate in later-line ADC monotherapy. The burgundy vertical line denotes the full-data primary GLMM estimate.

**Figure S4.** Leave-one-study-out analysis for grade ≥3 treatment-emergent adverse events in later-line ADC monotherapy. The burgundy vertical line denotes the full-data primary GLMM estimate.

**Figure S5.** Leave-one-study-out analysis for grade ≥3 treatment-related adverse events in later-line ADC monotherapy. The burgundy vertical line denotes the full-data primary GLMM estimate.
