# Supplementary material for: Antibody–drug conjugates in advanced small-cell lung cancer: a systematic review and single-arm meta-analysis of later-line monotherapy
Source: Front Immunol. 2026 Oct 7;17:1929911. doi: 10.3389/fimmu.2026.1929911 (PMC13683592; doi:10.3389/fimmu.2026.1929911)
Supplement: Supplementary file 2 [file Table2.docx]

#!/usr/bin/env Rscript

\# Reproducible analysis for the systematic review of antibody-drug conjugates

\# in advanced small-cell lung cancer. The script reads the submitted CSV file;

\# it does not rely on local PDF filenames or legacy spreadsheet calculations.

suppressPackageStartupMessages({

library(readr)

library(dplyr)

library(purrr)

library(stringr)

library(meta)

library(ggplot2)

})

options(stringsAsFactors = FALSE, scipen = 999)

set.seed(20260827)

script_arg <- grep("^--file=", commandArgs(trailingOnly = FALSE), value = TRUE)

script_path <- if (length(script_arg)) {

normalizePath(sub("^--file=", "", script_arg[[1]]), winslash = "/")

} else {

normalizePath("ADC_SCLC_meta_analysis.R", winslash = "/")

}

root <- dirname(script_path)

input_file <- file.path(root, "ADC_SCLC_arm_level_dataset.csv")

output_root <- file.path(root, "ADC_SCLC_analysis_output")

dir_results <- file.path(output_root, "results")

dir_figures <- file.path(output_root, "figures")

dir_qc <- file.path(output_root, "qc")

invisible(lapply(c(dir_results, dir_figures, dir_qc), dir.create,

recursive = TRUE, showWarnings = FALSE))

stop_with <- function(...) stop(sprintf(...), call. = FALSE)

if (!file.exists(input_file)) stop_with("Input file not found: %s", input_file)

dat <- read_csv(input_file, show_col_types = FALSE, na = c("", "NA")) %>%

mutate(

include_primary = as.logical(include_primary),

event = as.integer(event),

total = as.integer(total),

target = molecular_target

)

required_columns <- c(

"record_id", "supplement_record_id", "study_id", "author_year", "trial_id",

"arm_id", "regimen", "clinical_context", "therapy_type", "molecular_target",

"dose", "outcome", "outcome_definition", "event", "total", "analysis_set",

"include_primary", "analysis_role", "parent_record_id",

"source_citation_location", "provenance", "cohort_relation", "exclusion_reason"

)

missing_columns <- setdiff(required_columns, names(dat))

if (length(missing_columns)) {

stop_with("Missing required columns: %s", paste(missing_columns, collapse = ", "))

}

main_records <- dat %>% filter(analysis_role != "cohort_choice_sensitivity")

alternative_records <- dat %>% filter(analysis_role == "cohort_choice_sensitivity")

primary_data <- main_records %>% filter(include_primary)

checks <- tibble(

check = c(

"record_id is unique",

"event and denominator are non-missing integers",

"0 <= event <= denominator",

"molecular target is present for every row",

"source citation/location contains no local PDF filename",

"primary rows are later-line monotherapy",

"one primary record per trial and outcome",

"excluded main records have an exclusion reason",

"alternative cohorts identify a valid parent record",

"fixed-time PFS/OS values are not analysed as x/n"

),

pass = c(

!anyDuplicated(dat$record_id),

all(!is.na(dat$event) & !is.na(dat$total) & dat$event == floor(dat$event) & dat$total == floor(dat$total)),

all(dat$event >= 0 & dat$total > 0 & dat$event <= dat$total),

all(!is.na(dat$molecular_target) & dat$molecular_target != ""),

!any(str_detect(dat$source_citation_location, regex("(?:^|[;/ ])[^;/]*\\.pdf(?:$|[;/ ])", ignore_case = TRUE)), na.rm = TRUE),

all(primary_data$clinical_context == "Later-line monotherapy"),

!anyDuplicated(paste(primary_data$trial_id, primary_data$outcome, sep = "::")),

all(main_records$exclusion_reason[!main_records$include_primary] != ""),

all(alternative_records$parent_record_id %in% main_records$record_id),

!any(str_detect(main_records$outcome, regex("month|PFS|OS", ignore_case = TRUE)))

)

)

write_csv(checks, file.path(dir_qc, "data_validation.csv"))

if (!all(checks$pass)) {

stop_with("Data validation failed: %s", paste(checks$check[!checks$pass], collapse = "; "))

}

outcomes_for_pooling <- c(

"ORR", "DCR", "All-grade TEAE", "Grade >=3 TEAE", "Serious TEAE",

"All-grade TRAE", "Grade >=3 TRAE", "Serious TRAE"

)

fit_glmm <- function(x) {

if (nrow(x) < 3) return(NULL)

metaprop(

event = event, n = total, studlab = author_year, data = x,

sm = "PLOGIT", method = "GLMM", common = FALSE, random = TRUE,

prediction = TRUE, level = 0.95, level.predict = 0.95,

method.predict = "V", warn = TRUE

)

}

fit_iv <- function(x) {

if (nrow(x) < 3) return(NULL)

metaprop(

event = event, n = total, studlab = author_year, data = x,

sm = "PLOGIT", method = "Inverse", incr = 0.5,

method.incr = "only0", method.tau = "REML",

method.random.ci = "HK", common = FALSE, random = TRUE,

prediction = TRUE, level = 0.95, level.predict = 0.95,

method.predict = "V", warn = TRUE

)

}

back_transform <- function(x) plogis(x)

summarize_meta <- function(m, outcome, model_name, x) {

if (is.null(m)) return(tibble())

q_values <- as.numeric(m$Q)

q_p_values <- as.numeric(m$pval.Q)

tibble(

outcome = outcome,

model = model_name,

k = nrow(x),

participants = sum(x$total),

events = sum(x$event),

estimate = back_transform(m$TE.random),

ci_low = back_transform(m$lower.random),

ci_high = back_transform(m$upper.random),

pi_low = back_transform(m$lower.predict),

pi_high = back_transform(m$upper.predict),

tau2_logit = m$tau2,

tau_logit = m$tau,

I2 = m$I2,

Q = q_values[[1]],

Q_df = as.numeric(m$df.Q)[[1]],

Q_p = q_p_values[[1]],

Q_LRT = if (length(q_values) >= 2) q_values[[2]] else NA_real_,

Q_LRT_p = if (length(q_p_values) >= 2) q_p_values[[2]] else NA_real_

)

}

primary_by_outcome <- primary_data %>%

filter(outcome %in% outcomes_for_pooling) %>%

group_split(outcome) %>%

set_names(map_chr(., ~ unique(.x$outcome)))

models_glmm <- map(primary_by_outcome, fit_glmm)

models_iv <- map(primary_by_outcome, fit_iv)

summary_glmm <- imap_dfr(models_glmm, function(model, outcome) {

x <- primary_by_outcome[[outcome]]

summarize_meta(model, outcome,

"Primary: binomial-normal random-intercept GLMM (ML)", x)

})

summary_iv <- imap_dfr(models_iv, function(model, outcome) {

x <- primary_by_outcome[[outcome]]

summarize_meta(model, outcome,

"Sensitivity: inverse-variance logit, REML/Hartung-Knapp", x)

})

cohort_choice_data <- primary_data %>%

filter(outcome %in% c("ORR", "DCR"),

!record_id %in% alternative_records$parent_record_id) %>%

bind_rows(alternative_records %>% select(any_of(names(primary_data)))) %>%

arrange(outcome, author_year)

cohort_choice_by_outcome <- cohort_choice_data %>%

group_split(outcome) %>%

set_names(map_chr(., ~ unique(.x$outcome)))

cohort_choice_models <- map(cohort_choice_by_outcome, fit_glmm)

cohort_choice_summary <- imap_dfr(cohort_choice_models, function(model, outcome) {

x <- cohort_choice_by_outcome[[outcome]]

summarize_meta(model, outcome,

"Sensitivity: recommended/expansion efficacy cohorts, GLMM (ML)", x)

})

pooled_models <- bind_rows(summary_glmm, summary_iv, cohort_choice_summary)

write_csv(pooled_models, file.path(dir_results, "pooled_models.csv"))

write_csv(cohort_choice_data, file.path(dir_results, "cohort_choice_sensitivity_data.csv"), na = "")

exact_ci <- function(events, total) {

interval <- binom.test(events, total, conf.level = 0.95)$conf.int

c(interval[[1]], interval[[2]])

}

study_level <- imap_dfr(primary_by_outcome, function(x, outcome) {

intervals <- map2(x$event, x$total, exact_ci)

iv_model <- models_iv[[outcome]]

weights <- if (is.null(iv_model)) rep(NA_real_, nrow(x)) else iv_model$w.random / sum(iv_model$w.random) * 100

x %>% mutate(

estimate = event / total,

ci_low = map_dbl(intervals, 1),

ci_high = map_dbl(intervals, 2),

iv_sensitivity_weight_pct = weights

)

})

write_csv(study_level, file.path(dir_results, "study_level_effects.csv"), na = "")

leave_one_out <- imap_dfr(primary_by_outcome, function(x, outcome) {

if (nrow(x) < 4) return(tibble())

map_dfr(seq_len(nrow(x)), function(index) {

model <- fit_glmm(x[-index, , drop = FALSE])

tibble(

outcome = outcome,

omitted_study = x$author_year[[index]],

omitted_arm = x$regimen[[index]],

k_remaining = nrow(x) - 1,

estimate = back_transform(model$TE.random),

ci_low = back_transform(model$lower.random),

ci_high = back_transform(model$upper.random),

tau2_logit = model$tau2,

I2 = model$I2

)

})

})

write_csv(leave_one_out, file.path(dir_results, "leave_one_out_glmm.csv"))

context_descriptive <- main_records %>%

filter(outcome %in% c("ORR", "DCR", "Grade >=3 TEAE", "Grade >=3 TRAE", "Grade 3-4 TRAE")) %>%

mutate(

estimate = event / total,

interval = map2(event, total, exact_ci),

ci_low = map_dbl(interval, 1),

ci_high = map_dbl(interval, 2)

) %>%

select(-interval) %>%

arrange(outcome, clinical_context, author_year)

write_csv(context_descriptive, file.path(dir_results, "clinical_context_descriptive.csv"), na = "")

target_groups <- primary_data %>%

filter(outcome %in% c("ORR", "DCR")) %>%

group_by(outcome, molecular_target) %>%

group_split()

target_summary <- map_dfr(target_groups, function(x) {

if (nrow(x) < 3) {

return(tibble(

outcome = unique(x$outcome), molecular_target = unique(x$molecular_target),

k = nrow(x), participants = sum(x$total), events = sum(x$event),

estimate = NA_real_, ci_low = NA_real_, ci_high = NA_real_,

pi_low = NA_real_, pi_high = NA_real_, tau2_logit = NA_real_, I2 = NA_real_,

synthesis = "Not pooled (k < 3)"

))

}

model <- fit_glmm(x)

tibble(

outcome = unique(x$outcome), molecular_target = unique(x$molecular_target),

k = nrow(x), participants = sum(x$total), events = sum(x$event),

estimate = back_transform(model$TE.random),

ci_low = back_transform(model$lower.random),

ci_high = back_transform(model$upper.random),

pi_low = back_transform(model$lower.predict),

pi_high = back_transform(model$upper.predict),

tau2_logit = model$tau2, I2 = model$I2,

synthesis = "Exploratory GLMM; no between-target comparison"

)

})

write_csv(target_summary, file.path(dir_results, "exploratory_target_summaries.csv"), na = "")

format_pct <- function(x) sprintf("%.1f%%", 100 * x)

draw_forest <- function(outcome_name) {

x <- study_level %>%

filter(outcome == outcome_name) %>%

arrange(estimate) %>%

mutate(

y = seq_len(n()),

study_label = paste0(author_year, " | ", regimen, " (", dose, ")")

)

summary_row <- summary_glmm %>% filter(outcome == outcome_name)

if (!nrow(x) || !nrow(summary_row)) return(NULL)

y_breaks <- c(0, x$y)

y_labels <- c("Random-effects GLMM", x$study_label)

ggplot(x, aes(x = estimate, y = y)) +

geom_vline(xintercept = summary_row$estimate, color = "#B22222", linewidth = 0.45) +

geom_errorbarh(aes(xmin = ci_low, xmax = ci_high), height = 0.13,

color = "#1B4F72", linewidth = 0.55) +

geom_point(aes(size = iv_sensitivity_weight_pct), shape = 15, color = "#1B4F72") +

annotate("segment", x = summary_row$ci_low, xend = summary_row$ci_high,

y = 0, yend = 0, color = "#B22222", linewidth = 1.1) +

annotate("point", x = summary_row$estimate, y = 0, shape = 18,

size = 4.0, color = "#B22222") +

scale_x_continuous(labels = scales::percent_format(accuracy = 1),

limits = c(0, 1), breaks = seq(0, 1, 0.2)) +

scale_y_continuous(breaks = y_breaks, labels = y_labels,

expand = expansion(add = c(0.55, 0.45))) +

scale_size_area(max_size = 4.2, guide = "none") +

labs(x = "Proportion (95% CI)", y = NULL) +

theme_classic(base_family = "Arial", base_size = 8) +

theme(

axis.text.y = element_text(size = 7, color = "#222222"),

axis.text.x = element_text(size = 7, color = "#222222"),

axis.title.x = element_text(size = 8),

panel.grid.major.x = element_line(color = "#E5E7EB", linewidth = 0.35),

plot.margin = margin(5, 5, 5, 5)

)

}

figure_specs <- tibble::tribble(

~stem, ~outcome,

"Figure_2_ORR_later_line_monotherapy", "ORR",

"Figure_3_DCR_later_line_monotherapy", "DCR",

"Figure_4_grade3_TEAE_later_line_monotherapy", "Grade >=3 TEAE",

"Figure_5_grade3_TRAE_later_line_monotherapy", "Grade >=3 TRAE"

)

pwalk(figure_specs, function(stem, outcome) {

plot <- draw_forest(outcome)

if (is.null(plot)) return(invisible(NULL))

n_studies <- nrow(study_level %>% filter(.data$outcome == .env$outcome))

height_mm <- max(72, 35 + 6.5 * n_studies)

ggsave(file.path(dir_figures, paste0(stem, ".pdf")), plot,

width = 183, height = height_mm, units = "mm", device = cairo_pdf)

ggsave(file.path(dir_figures, paste0(stem, ".tiff")), plot,

width = 183, height = height_mm, units = "mm", dpi = 600,

device = "tiff", compression = "lzw", bg = "white")

ggsave(file.path(dir_figures, paste0(stem, ".png")), plot,

width = 183, height = height_mm, units = "mm", dpi = 300, bg = "white")

})

text_results <- summary_glmm %>%

filter(outcome %in% c("ORR", "DCR", "Grade >=3 TEAE", "Grade >=3 TRAE")) %>%

transmute(

sentence = sprintf(

"%s: k=%d, %d/%d events, pooled proportion %s (95%% CI %s-%s), tau^2=%.3f, I^2=%.1f%%, 95%% prediction interval %s-%s.",

outcome, k, events, participants, format_pct(estimate), format_pct(ci_low),

format_pct(ci_high), tau2_logit, 100 * I2, format_pct(pi_low), format_pct(pi_high)

)

)

write_lines(text_results$sentence, file.path(dir_results, "text_ready_primary_results.txt"))

writeLines(capture.output(sessionInfo()), file.path(dir_qc, "sessionInfo.txt"), useBytes = TRUE)

message("Analysis completed successfully. Outputs written to: ", output_root)
